# A membrane-bound ankyrin repeat protein confers race-specific leaf rust disease resistance in wheat

**DOI:** 10.1038/s41467-020-20777-x

**Published:** 2021-02-11

**Authors:** Markus C. Kolodziej, Jyoti Singla, Javier Sánchez-Martín, Helen Zbinden, Hana Šimková, Miroslava Karafiátová, Jaroslav Doležel, Julien Gronnier, Manuel Poretti, Gaétan Glauser, Wangsheng Zhu, Philipp Köster, Cyril Zipfel, Thomas Wicker, Simon G. Krattinger, Beat Keller

**Affiliations:** 1 University of Zurich, Department of Plant and Microbial Biology, Zollikerstrasse 107, 8008 Zurich, Switzerland; 2 Institute of Experimental Botany of the Czech Academy of Sciences, Centre of the Region Hana for Biotechnological and Agricultural Research, Šlechtitelů 31, 779 00 Olomouc, Czech Republic; 3 Neuchâtel Platform of Analytical Chemistry, Université de Neuchâtel, Avenue de Bellevaux 51, 2000 Neuchâtel, Switzerland; 4 Department of Molecular Biology, Max Planck Institute for Developmental Biology, 72076 Tübingen, Germany; 5 College of Plant Protection, China Agricultural University, 100193 Beijing, China; 6 King Abdullah University of Science and Technology (KAUST), Biological and Environmental Science and Engineering Division (BESE), Thuwal, 23955-6900 Kingdom of Saudi Arabia

**Keywords:** Agricultural genetics, Plant genetics, Plant immunity, Biotic

## Abstract

Plasma membrane-associated and intracellular proteins and protein complexes play a pivotal role in pathogen recognition and disease resistance signaling in plants and animals. The two predominant protein families perceiving plant pathogens are receptor-like kinases and nucleotide binding-leucine-rich repeat receptors (NLR), which often confer race-specific resistance. Leaf rust is one of the most prevalent and most devastating wheat diseases. Here, we clone the race-specific leaf rust resistance gene *Lr14a* from hexaploid wheat. The cloning of *Lr14a* is aided by the recently published genome assembly of Arina*LrFor*, an *Lr14a*-containing wheat line. *Lr14a* encodes a membrane-localized protein containing twelve ankyrin (ANK) repeats and structural similarities to Ca^2+^-permeable non-selective cation channels. Transcriptome analyses reveal an induction of genes associated with calcium ion binding in the presence of *Lr14a*. Haplotype analyses indicate that *Lr14a-*containing chromosome segments were introgressed multiple times into the bread wheat gene pool, but we find no variation in the *Lr14a* coding sequence itself. Our work demonstrates the involvement of an ANK-transmembrane (TM)-like type of gene family in race-specific disease resistance in wheat. This forms the basis to explore ANK-TM-like genes in disease resistance breeding.

## Abstract (web summary)

Winter wheat cultivar Forno harbors a race-specific leaf rust resistance locus *Lr14a*, but the causative gene is unknown. Here, the authors show that *Lr14a* encodes a membrane-localized protein containing ankyrin repeats and *Lr14a*-containing segments have been introgressed into the bread wheat gene pool multiple times.

## Introduction

Leaf rust, caused by the fungal pathogen *Puccinia triticina*, is one of the most damaging wheat diseases^1^. Long-distance dispersal of *P. triticina* spores by wind facilitates the rapid spread of newly evolved leaf rust pathogen races^1^. Genetic resistance within the wheat gene pool is the most sustainable way to control leaf rust^1^. Some wheat cultivars are characterized by exceptional levels of leaf rust resistance that continues to be effective in the field even after decades. Such durable and broad-spectrum resistance is often the result of multiple, additively acting quantitative disease resistance genes. For instance, the Swiss winter wheat cultivar Forno (released in the 1980s) continues to show near immunity to all tested *P. triticina* races. The durable leaf rust resistance in Forno is controlled by several genes with additive effect^2^, including the adult plant resistance genes *Lr34*^3^ and *Lr75*^4^, and a race-specific all-stage resistance locus on chromosome arm 7BL that was postulated to be *Lr14a*^2,4^. Of these three genes, only *Lr14a* confers seedling resistance at ambient temperatures. A genetic interaction of *Lr14a* with *Lr75*^2^ and a characteristic mesothetic seedling resistance phenotype with both fully developed leaf rust uredia and hypersensitive flecks occurring on the same leaf^5^ were observed. Earlier work on *Lr14a* has shown a strong dependence on environmental and genetic factors^5–8^. These unique properties of phenotypic and genetic interactions of a major seedling disease resistance gene make *Lr14a* an interesting target for molecular functional analyses (Supplementary Note 1).

Here, we use two Forno-derived backcross lines, Arina*LrFor*^4^ *(Lr75*+*Lr14a* in the genetic background of the susceptible Swiss winter wheat cultivar Arina) and Arina*Lr14a*, to isolate *Lr14a*. It encodes LR14A, an ankyrin repeat containing protein located in the plasma membrane and possibly acting as a non-selective, Ca^2+^-permeable cation channel to mediate resistance.

## Results and discussion

### *Lr14a* confers leaf rust resistance in Arina*LrFor*

**Fig. 1:**
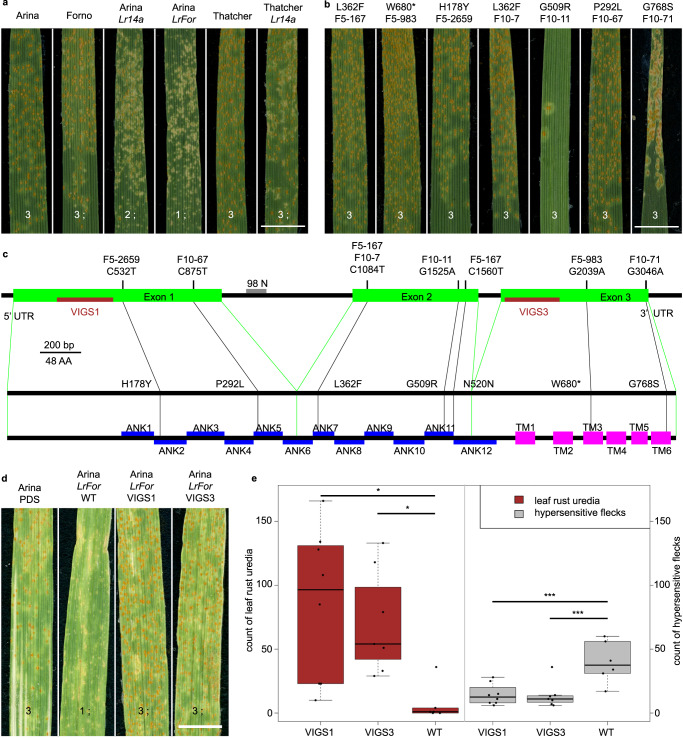
*Lr14a* encodes an ANK-TM protein. **a** Seedling infections of wheat genotypes Arina, Forno, Arina*Lr14a*, Arina*LrFor*, Thatcher, and Thatcher*Lr14a*. **b** Seedling infections of Arina*LrFor-*derived EMS mutants. Scale bars = 0.5 cm. **c** Gene and protein structures of *Lr14a*. *Lr14a* consists of three exons (green). The first intron contains a repetitive microsatellite, of which 98 bp (gray, N) could not be sequenced. A 273 bp and 267 bp long sequence were targeted in exons 1 and 3, respectively (brown) for virus-induced gene silencing (VIGS). The first four non-synonymous mutations are located in the N-terminal ankyrin repeat (ANK) domain (blue) of the protein, together with a silent mutation. The premature stop codon and the fifth non-synonymous mutation are located within the predicted transmembrane domains (TM) (magenta) at the C-terminus of the protein. **d** Representative images showing increased leaf rust susceptibility after silencing the *Lr14a* candidate gene through VIGS. PDS = phytoene desaturase control, WT = barley stripe mosaic virus without silencing construct. Scale bar = 0.7 cm. **e** Quantification of leaf rust symptoms. The numbers of leaf rust uredia (brown) and hypersensitive flecks (gray) were determined. Squares of 0.5 × 2 cm were counted in the middle of representative leaves from one experiment VIGS1 = 8 leaves, VIGS3 = 7 leaves, WT = 6 leaves. Statistics: for both datasets, a Levene test was performed, followed by ANOVA and a Shapiro-Wilk test for leaf rust uredia and Tukey-HSD for hypersensitive flecks. For boxplots: minimum value = lower whisker, maximum value = upper whisker, median = middle value of box, lower quartile = median of lower half of dataset, upper quartile = median of upper half of dataset, datapoint outside of whiskers = potential outlier. * = *p*-value < 0.05, *** = *p*-value < 0.001. Leaf rust infection types in (**a**), (**b**), and (**d**) are as follows: 1 = small uredia with necrosis, 2 = small to medium sized uredia with necrosis or chlorosis, 3 = medium sized uredia with or without chlorosis, ; = hypersensitive flecks. Source data underlying (**a**), (**b**), (**d**), and (**e**) are provided as a Source Data file.

Both backcross lines and Thatcher*Lr14a* (*Lr14a* from the Canadian wheat cultivar Selkirk introgressed into the susceptible background of Thatcher)^7^ showed an *Lr14a*-characteristic mesothetic resistance response^5^ at the seedling stage (Fig. 1a). F_1_ plants from a cross between Arina and Arina*Lr14a* were resistant, indicating that the *Lr14a*-mediated resistance is dominant (Supplementary Fig. 1a). Eleven tested *P. triticina* isolates produced identical avirulence/virulence formulae on Arina*LrFor*, Arina*Lr14a*, and Thatcher*Lr14a*, corroborating that the gene on chromosome 7BL of Arina*LrFor* is *Lr14a* (Supplementary Table 1). Adult Arina*LrFor* and Arina*Lr14a* plants showed higher resistance compared to the susceptible parent Arina in the field (Supplementary Fig. 1b, c).

### EMS mutants and VIGS identify and validate *Lr14a*

To clone *Lr14a*, we identified seven seedling-susceptible mutant lines from two independent Arina*LrFor*-derived ethyl methanesulfonate (EMS) mutant populations (Fig. 1b). Chromosome 7B of three of these mutants was isolated by flow cytometry and sequenced by short-read sequencing. Reads were aligned against the recently published chromosome-scale genome assembly of Arina*LrFor*^9^. To identify candidate genes, the Arina*LrFor* assembly was fragmented in silico and subjected to the MutChromSeq pipeline^10^. This resulted in the identification of a single gene-containing contig, in which all three mutant lines showed independent EMS-induced single nucleotide polymorphisms (SNPs) (G/C to A/T) compared to the wild-type in the predicted coding sequence (Fig. 1c). Amplification and sequencing revealed additional non-synonymous SNPs in the four remaining mutants (Fig. 1c). To functionally validate the candidate gene, we performed virus-induced gene silencing (VIGS) by targeting two gene-specific sequences (Fig. 1c). Silencing of *Lr14a* in Arina*LrFor* plants resulted in increased susceptibility apparent by increased numbers of leaf rust uredia and decreased numbers of hypersensitive flecks (Fig. 1d, e).

### *Lr14a* encodes an ankyrin-transmembrane protein

The *Lr14a* coding sequence is 2340 bp long, consists of three exons, and translates into a predicted 779 amino acid protein with an N-terminal domain, containing 12 ankyrin (ANK) repeats, followed by six predicted transmembrane (TM) helices (Fig. 1c, Supplementary Fig. 2a–d). ANK repeat proteins have been described in archaea, bacteria, viruses, and eukaryots^11^. The ANK repeat domain consists of up to 30 repeats of a 33-amino acid motif, which forms two α-helices. ANK repeat domain-containing proteins either have the ANK repeat domain alone or in combination with various other domains, including TM domains, calmodulin binding domains, zinc finger domains, K^+^ channel domains, and many more^12^. Several ANK proteins in plants play important roles in plant immunity, development, and growth, and they are typically involved in protein-protein interactions^12,13^. No signal peptide was predicted in the N-terminus of the LR14A protein and no alternative splicing was found. Two of the EMS mutants had SNPs in the sequence encoding the predicted TM domain, resulting in an amino acid substitution and in a premature stop codon, respectively. The remaining five mutants carry SNPs, resulting in amino acid substitutions in the predicted α-helices that form the structural backbone of the ANK repeats. An identical amino acid substitution (L362F) was recovered from the two independent EMS populations. (Fig. 1c, Supplementary Fig. 2e).

### *Lr14a*-specific diagnostic marker

An *Lr14a*-specific presence/absence marker, targeting the first exon of the gene (Supplementary Fig. 3a) was genetically mapped to the *Lr14a* region on chromosome arm 7BL (Supplementary Fig. 3b, Supplementary Note 1) in a bi-parental mapping population and showed complete co-segregation with the *Lr14a*-mediated rust resistance (Supplementary Data 1). The marker also amplified in the wheat lines Hope, Kalyansona, and Selkirk, which have been described as donor lines of *Lr14a* in wheat breeding^5^. In summary, our results from the mutagenesis experiment (Fig. 1b), genetic mapping (Supplementary Fig. 3b), and VIGS (Fig. 1d,e) demonstrate that *Lr14a* encodes an ANK-TM protein.

### *Lr14a* phenotype is genotype and environment dependent

Within the bi-parental mapping population, *Lr14a-*containing lines showed a broad range of mesothetic resistance responses with varying proportions of hypersensitive flecks and leaf rust uredia. Differences in the magnitude of the *Lr14a*-mediated resistance response were also observed in various *Lr14a*-containing wheat cultivars (Supplementary Fig. 3c). This is possibly the result of genetic modifiers, which have previously been described for *Lr14a*^5,7,8^ (Supplementary Note 1). The strong variability is reminiscent of quantitatively acting disease resistance genes^14^. Furthermore, the *Lr14a* gene action has been described as temperature sensitive^6^. Inoculation experiments at different temperatures were in agreement with previous reports^12^ and *Lr14a* was more effective at lower temperatures (Supplementary Fig. 4a).

### Race-specific induction of *Lr14a* expression

**Fig. 2:**
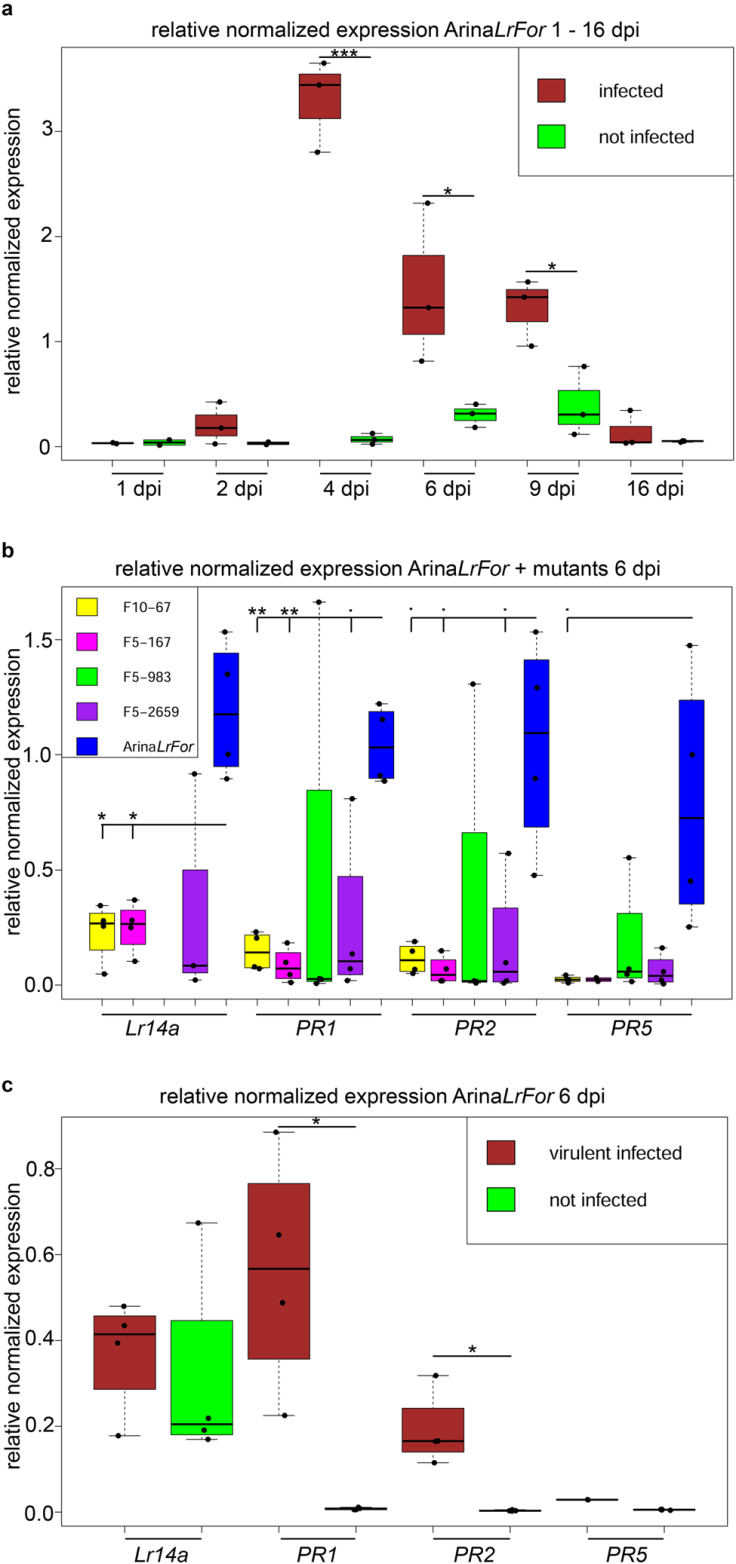
Relative normalized expression of *Lr14a* and pathogenesis-related marker genes in different conditions. **a**, *Lr14a* expression in Arina*LrFor* with and without infection (brown/green) with the avirulent *Puccinia triticina* isolate 96209. *n* = 3. Statistics: Shapiro-Wilk test for each dataset was followed by F-test for each timepoint comparison. 16 dpi was analyzed with an unpaired two-sided Wilcox test (*p*-value =0.7). 1, 2, 4, 6, 9 dpi were analyzed with a one-sided t-test. *p*-values = 0.5861, 0.161, 0.0001153, 0.02835, 0.01289, respectively. **b** *Lr14a* expression in Arina*LrFor* was higher than expression in *Lr14a* EMS mutants (green one below qPCR detection limit), while pathogenesis-related (*PR*) marker expression in Arina*LrFor* (blue) was higher than in the *Lr14a* EMS mutant lines (yellow, magenta, green, violet), 6 dpi with avirulent *P. triticina* isolate 96209. *n* = 4. Statistics: Datasets of different gene expressions were analyzed by a Levene test, followed by a Welch-ANOVA one-way test for *Lr14a*, *PR1,* and *PR2* and a Kruskal–Wallis test for *PR5*. **c** *Lr14a* and *PR* marker expression of Arina*LrFor*, 6 dpi with virulent *P. triticina* isolate 95037 (brown) and not infected (green). *Lr14a* transcript levels showed no significant differences between non-infected leaves and after inoculation with a virulent isolate. Two *PR* genes were activated after infection with the virulent pathogen, while *PR5* showed no detectable induction under the same conditions. n = 4. Statistics: Shapiro–Wilk tests for each dataset were followed by F-test for each gene expression comparison. Wilcox test, two-sided was used for *Lr14a* (*p*-value = 0.6857), Welch test, two-sided was used for *PR1* (*p*-value = 0.02815) and *PR2* (*p*-value = 0.02377), *PR5* was excluded from statistics because the infected condition has only two datapoints. For boxplots in (**a**), (**b**), (**c**): minimum value = lower whisker, maximum value = upper whisker, median = middle value of box, lower quartile = median of lower half of dataset, upper quartile = median of upper half of dataset, datapoint outside of whiskers = potential outlier. · = *p*-value < 0.1, * = *p*-value < 0.05, ** = *p*-value < 0.01, *** = *p*-value < 0.001. Source data are provided as a Source Data file.

*Lr14a* expression was very low or undetectable in uninfected plants but increased two days post-inoculation (dpi) with the avirulent *P. triticina* isolate 96209. Expression peaked around four dpi, and then decreased again (Fig. 2a). In the susceptible EMS mutants, *Lr14a* expression levels were lower compared to Arina*LrFor* six dpi (Fig. 2b). The increase in *Lr14a* transcript levels correlated with an induction of pathogenesis-related (*PR*) genes^15^ (Fig. 2b). Arina*LrFor* seedlings inoculated with a virulent *P. triticina* isolate showed very low or undetectable *Lr14a* transcript levels similar as non-inoculated plants. In these plants, two of three *PR* genes showed a higher induction compared to non-infected plants while the induction of *PR5* seems to be dependent on an *Lr14a*-specific mechanism (Fig. 2c). These results show that the *Lr14a* gene is specifically expressed after inoculation with an avirulent *P. triticina* isolate, resulting in race-specific resistance.

### *Lr14a* is highly conserved in the gene pool of grasses

We used the *Lr14a-*specific marker to assess the distribution of *Lr14a* in a global bread wheat collection (Supplementary Data 2). Out of 99 tested cultivars, 27 contained *Lr14a*. The *Lr14a*-positive lines showed different resistance responses, probably as a result of genetic modifiers^5,7,8^ (Supplementary Fig. 3c). The other 72 cultivars had a null-allele for the marker, indicating the absence of the gene. In all 27 wheat lines that were positive for the *Lr14a*-derived marker, the *Lr14a* coding sequences were identical to the one found in Arina*LrFor*. Two previously described alleles of *Lr14a*, named *Lr14b*^7^ and *Lr14c*^16^ were investigated as well. The *Lr14a*-derived marker failed to produce an amplification product in Thatcher*Lr14b*. On the other hand, the marker amplified a product in the *Lr14*c-containing durum wheat line Creso. Amplification of the full-length coding sequence from Creso revealed 100% sequence identity to *Lr14a*. We therefore concluded that *Lr14b* is not a true allele of *Lr14a* and that a modifier of resistance resulted in the misinterpretation of an *Lr14c* allele (Supplementary Note 1). We extended our marker analysis to spelt (*Triticum aestivum* ssp*. spelta*), tetraploid wheat, and wild wheat relatives aiming to identify *Lr14a* diversity. Seven spelt lines (Supplementary Note 2) and 41 tetraploid Turkish durum cultivars and landraces, amongst them Hevidi (WW198) and Menceki (WW200) (Supplementary Fig. 3f), were positive for the *Lr14a*-specific marker (Supplementary Fig. 3a, Supplementary Data 2). The coding sequences of *Lr14a* were identical in all marker-positive spelt and durum wheat genotypes, suggesting a high degree of sequence conservation across different wheat subspecies. *Lr14a*-containing spelt lines showed no resistance response when infected with the avirulent *P. triticina* isolate 96209 (Supplementary Fig. 3d). However, crosses between three of these spelt lines and the susceptible bread wheat cultivar Arina produced F_1_ plants with stronger seedling resistance (including mesothetic responses) than either parent after inoculation with *P. triticina* isolate 96209 (Supplementary Fig. 3e), providing evidence for the presence of *Lr14a* modifiers^5,7,8^ (Supplementary Note 2).

To further study the origin of *Lr14a*, we analyzed collections comprising of 266 wild emmer wheat (*Triticum turgidum* ssp. *diccocoides*) accessions^17^ from Israel, Lebanon, and Turkey (Supplementary Data 2). The *Lr14a-*derived marker amplified in only eight wild emmer accessions collected near the Turkish cities of Gaziantep and Siverek close to the Turkish-Syrian border. Again, the *Lr14a* coding sequence was identical with *Lr14a* from Arina*LrFor* in all the eight wild emmer accessions. These results indicate that *Lr14a* is rare in the wild emmer gene pool and only present in accessions from a particular geographic region. The absence of sequence diversity in wild emmer wheat, cultivated durum wheat landraces and hexaploid wheat suggests a low selection pressure in recent evolution which might possibly point to an indirect interaction of LR14A and the corresponding avirulence gene product from the leaf rust pathogen.

*Lr14a’s* origin in the hexaploid bread wheat gene pool was traced back to a cross made in 1916 in South Dakota between the tetraploid, cultivated Yaroslav emmer and the bread wheat cultivar Marquis^5,18^, which resulted in the famous bread wheat cultivar Hope. Yaroslav emmer and Hope were both positive for the *Lr14a*-gene-specific marker, showed a mesothetic resistance phenotype (Supplementary Fig. 3a, c), and their *Lr14a* coding sequences were identical to the sequence in Arina*LrFor*. The origin of *Lr14a* in spelt is most likely different from the Yaroslav introgression because spelt and bread wheat separated thousands of years ago.

**Fig. 3:**
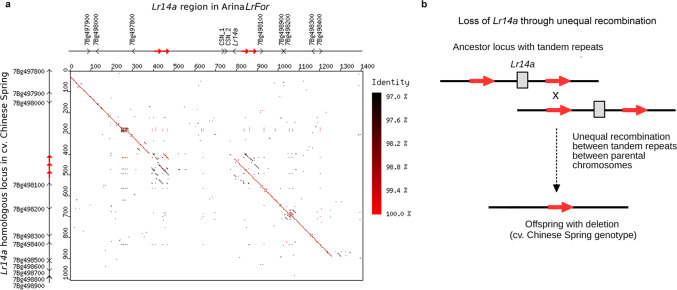
Sequence comparison of the *Lr14a* locus in wheat cultivars Arina*LrFor* and Chinese Spring. **a** Dotplot alignment of the *Lr14a* locus from Arina*LrFor* (horizontal) and Chinese Spring (vertical). Sequence identity is color coded according to the scale at the right. Sequences are annotated with genes depicted as arrow heads to indicate transcriptional orientation and tandem repeats are depicted as red arrows. **b** Model for tandem repeats flanking the region containing *Lr14a* and their function as templates for unequal crossing-over that led to the deletion of the *Lr14a* gene in the Chinese Spring haplotype.

Multiple independent origins of *Lr14a* in the hexaploid wheat gene pool were also supported by analyzing the *Lr14a* locus in multiple recently published wheat genomes^9^. We found *Lr14a* in the genomes of bread wheat cultivar Lancer and spelt accession PI190962^9^. Analyses of ~60 kb of flanking regions indicated that these represent different haplotypes, which diverged from the Arina*LrFor* haplotype approximately 44,000 and 10,000 years ago, respectively (Supplementary Note 3). Additionally, Chinese Spring contained an *Lr14a*-like haplotype, but lacked a ~315 kb region containing *Lr14a*, probably as a result of a deletion caused by unequal crossing over between large (10–50 kb) tandem repeats flanking the segment (Fig. 3, Supplementary Fig. 5, Supplementary Note 3, Supplementary Table 2).

### The ANK-TM protein LR14A might act as Ca^2+^ channel

The ANK-TM gene family contains 15 members in maize, 37 members in rice, and 40 members in the model plant *Arabidopsis thaliana*^13^. One of the ANK-TM genes in Arabidopsis is *Accelerated Cell Death 6* (*ACD6*) (AT4G14400), which has the same domain composition and shows 37% similarity to LR14A at the protein level (Supplementary Fig. 6a, b). LR14A protein homologs were described to be involved in quantitative disease resistance and stress response in Arabidopsis^19–21^ and most recently in maize^22^. Although all these proteins have the same ANK-TM domain structure, they share only a little sequence similarity with LR14A (Supplementary Fig. 6a, b). Phylogenetic analysis showed that the predicted LR14A protein is a member of a clade that has no counterpart in Arabidopsis (Supplementary Fig. 6c). Even within wheat, LR14A is unique and its closest homologs in Arina*LrFor* and Chinese Spring only have a 75% amino acid similarity. The closest Chinese Spring homolog is located on chromosome arm 5BL in a cluster with two other ANK-TM genes. Together with the low frequency and absence of polymorphisms in the *Lr14a* gene in tetraploid wild emmer wheat, this indicates a single introgression event in this species. However, the presence of sequence differences surrounding the gene suggests that *Lr14a* may have been subsequently introgressed multiple times into the hexaploid wheat gene pool.

**Fig. 4:**
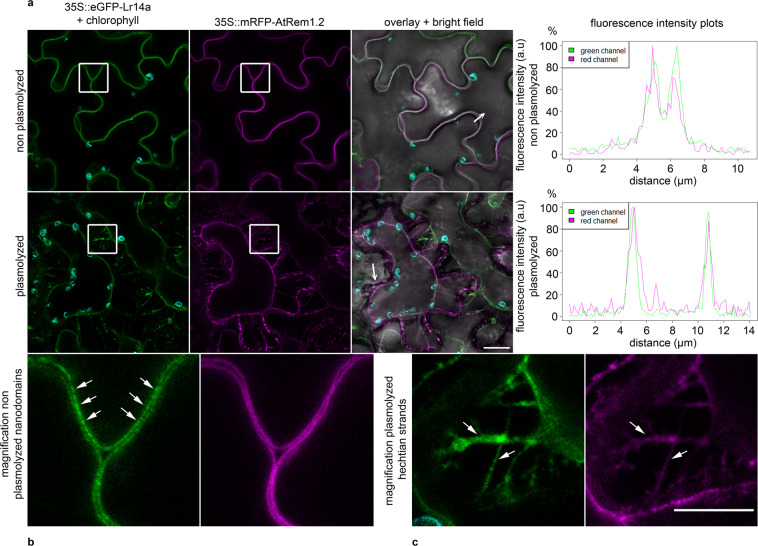
LR14A localized to the plasma membrane. *Nicotiana benthamiana* leaves transiently transformed with *Agrobacterium tumefaciens* carrying an overexpression construct with enhanced green fluorescence protein (eGFP) tagged LR14A and monomeric red fluorescence protein mRFP tagged AtREM1.2, a plasma membrane (PM) marker^63^. Green pictures show the eGFP fluorescence as well as chlorophyll (turquoise). Magenta pictures show mRFP fluorescence. All fluorescence channels are overlaid in a bright field (BF) picture together with turquoise chloroplast autofluorescence. Non-plasmolyzed lower leaf surface cells showed co-localization of both proteins in the plasma membrane. The LR14A protein accumulated in nanodomains visible by different intensity of spots on the PM (see also magnification). Plasmolyzed cells of lower leaf surface cells showed the localization of the LR14A protein on the Hechtian strands (also see magnification). Hechtian strands are connections between PM and cell wall and an indication for a PM localization of the LR14A protein^77^. Fluorescence intensity (a. u = arbitrary unit) plots show co-localization of fluorescence signal (fluorescence intensity is expressed in % of the maximum of fluorescence intensity of each channel) of eGFP and mRFP channel on not plasmolyzed PM and Hechtian strands over a 11 µm or 14 µm distance, respectively. Scale bar = 20 µm, for magnified images 10 µm. Source data are provided as a Source Data file.

**Fig. 5:**
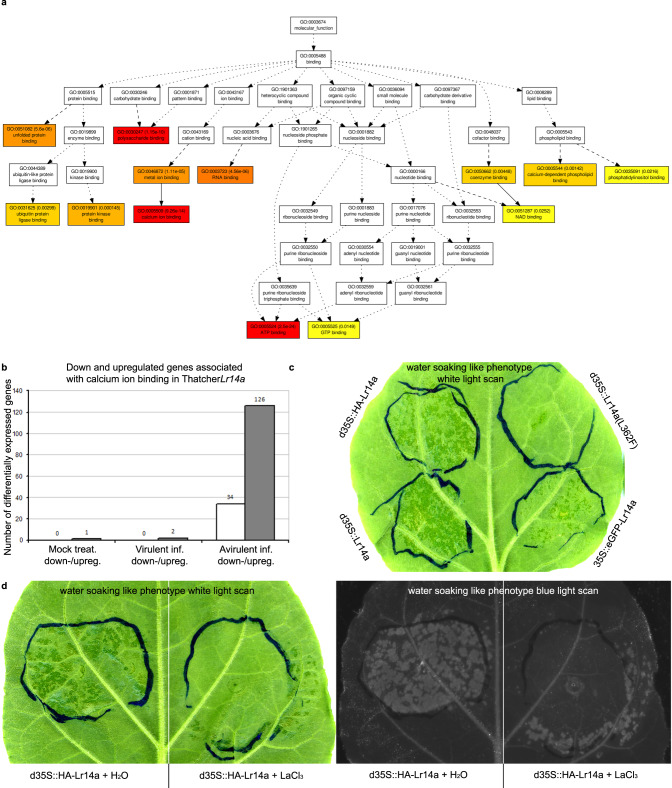
LR14A induces calcium ion binding associated genes in wheat and *Nicotiana benthamiana*. **a** Gene ontology (GO) network graph showing that *Lr14a* causes induction of calcium ion binding associated genes 8 days post-inoculation (dpi) in the wheat line Thatcher*Lr14a* compared to Thatcher after inoculation with an avirulent *P. triticina* isolate. **b** Number of differentially expressed genes that are associated with the GO term “calcium ion binding” in Thatcher*Lr14a* compared to Thatcher after mock treatment and inoculation with a virulent and avirulent *P. triticina* isolate, respectively. **c** *N. benthamiana* leaves transiently transformed with *Lr14a* coding sequences representing wild-type (WT) *Lr14a* coding sequence, an EMS loss-of-function mutant (L362F) *Lr14a* coding sequence, an N-terminal enhanced green fluorescence protein (eGFP) tagged *Lr14a* coding sequence version, and an N-terminal human influenza hemagglutinin (HA) tagged *Lr14a* coding sequence. The functional *Lr14a* versions induced a water-soaking^33^-like phenotype on the lower leaf surface, 3 dpi. This phenotype was weaker in the eGFP tagged version, possibly due to steric hindrance of the LR14A function or differences in the expression associated with different vector systems. The LR14A loss of function mutant (L362F) lost its ability to cause the water-soaking-like phenotype in *N. benthamiana*. **d** The water-soaking-like phenotype on the lower leaf surface of *N. benthamiana* caused by *Lr14a* overexpression 2 dpi was suppressed by infiltration of 2 mM Lanthanum(III) chloride (LaCl_3_), a calcium channel blocker^34,35^ two hours after Agrobacterium infiltration. The region infiltrated with HA-tagged *Lr14a* coding sequence and H_2_O as control showed a water-soaking-like phenotype, while the region infiltrated with *Lr14a* and LaCl_3_ showed an inhibition of this phenotype. The water-soaking-like phenotype is also detectable under blue light illumination. Source data underlying (**c**) and (**d**) are provided as a Source Data file.

Functional analyses of plant ANK-TM genes have mainly focused on *ACD6*, a key regulator of fitness tradeoffs between vegetative growth and general pathogen defense^23^. The gain of function mutant *acd6-1* has been reported to confer broad-spectrum disease resistance. In contrast to the broad spectrum resistance of the autoactive *acd6-1* with associated severe negative growth effects in Arabidopsis, *Lr14a* has a race-specific action and no *Lr14a*-associated negative effects on plant vigor were found by measuring adult plant height and seedling above-ground weight (Supplementary Fig. 4b–d). This is possibly the result of the absence of *Lr14a* expression in the absence of avirulent leaf rust pathogen races (Fig. 2a, c). Located in the plasma membrane^24^, ACD6 forms large complexes with other proteins in the membrane^25^ and shows interactions with pattern recognition receptors like BAK1 and CERK1^26^. Overexpression of N-terminal enhanced green fluorescence protein (eGFP) tagged LR14A in *Nicotiana benthamiana* epidermal cells revealed a plasma membrane localization of LR14A (Fig. 4). *ACD6* expression is regulated by salicylic acid (SA) levels, and ACD6 regulates SA accumulation and signaling, particularly in the *acd6-1* dominant gain-of-function mutant, which shows enhanced resistance to pathogens, amongst others due to elevated levels of SA^24,25,27^. We found no difference in salicylic acid (SA) levels between *P. triticina* inoculated Arina*LrFor* plants and the EMS-induced mutants (Supplementary Fig. 1d), suggesting that LR14A might act differently from ACD6. Interestingly, we identified a strong structural similarity between LR14A and calcium channels, including the transient receptor potential cation channel protein TRPA1^28^ (Supplementary Table 3). These results indicate that LR14A might function through an unknown resistance mechanism, possibly acting as a non-selective, Ca^2+^-permeable cation channel. To get further insight into the possible function of *Lr14a*, we performed an RNAseq experiment in Thatcher and Thatcher*Lr14a*. Thatcher*Lr14a* infected with an avirulent leaf rust pathogen isolate showed 7,986 differentially expressed genes compared to Thatcher 8 dpi and there was a significant differential expression for 160 genes associated with “calcium ion binding” (Fig. 5a, b, Supplementary Data 3,4,5). This reaction only occurred in Thatcher*Lr14a* infected with an avirulent *P. triticina* isolate. Inoculation of Thatcher*Lr14a* with the virulent *P. triticina* isolate 95037 and mock inoculations resulted in a very low number of differentially expressed genes (65 and 193, respectively) and there were only 2 and 1 differentially expressed genes, respectively, associated with calcium ion binding (Fig. 5b). To further corroborate *Lr14a*’s possible role in calcium fluxes, we transiently infiltrated *Lr14a* into *N. benthamiana* leaves and analyzed the differentially expressed genes 27 h post-infiltration (Supplementary Fig. 7). Similar to wheat, we observed a significant enrichment for 26 genes associated with “calcium ion binding” in *N. benthamiana* leaves infiltrated with *Lr14a* compared to empty vector controls (Supplementary Data 6,7,8). Five of these genes were related to the *A. thaliana* homologous protein At5g02490 encoding a HEAT SHOCK COGNATE PROTEIN, 70-2 (HSC70-2), which has been described as a development and stress regulator^29^. Another 11 up-regulated genes are homologs of different CALMODULIN LIKE proteins (CMLs), which are calcium-sensing proteins involved in intracellular signaling^30^. Calcium is a known secondary messenger in plants involved in diverse signaling processes, including hormone regulation, abiotic, and biotic stress response^31,32^. The gene expression analysis suggests that *Lr14a*, which is activated in wheat specifically after infection by an avirulent *P. triticina* isolate, is inducing genes involved in calcium signaling. The observation that infiltration of *Lr14a* alone results in similar changes of expression patterns in *N. benthamiana* further indicates that expression of *Lr14a* is sufficient for the induction of the observed changes in gene expression.

In *N. benthamiana* leaves, we observed a specific, *Lr14a*-dependent phenotype reminiscent to the previously described water-soaking^33^ phenotype. This phenotype is presumably caused by a change in the osmotic balance of leaf cells^33^. The same phenotype was also observed with an N-terminal human influenza hemagglutinin (HA) tagged *Lr14a* coding sequence as well as with an N-terminal eGFP tagged *Lr14a* coding sequence (Fig. 5c). The eGFP tagged phenotype was weaker, possibly due to the larger tag or differences in expression associated with different vector systems. Infiltration of the *Lr14a* coding sequence of the EMS loss-of-function mutant (L362F) did not result in the water-soaking-like phenotype (Fig. 5c). Based on the RNAseq data we suggest LR14A directly or indirectly induces changes in calcium ion fluxes at the plasma membrane. Overexpression of *Lr14a* in *N. benthamiana* could thus result in an osmotic imbalance, leading to the water-soaking-like phenotype (Fig. 5c). The water-soaking-like phenotype was abolished when Lanthanum(III) chloride (LaCl_3_)^34,35^ was infiltrated two hours after infiltration with *Lr14a* (Fig. 5d). LaCl_3_ is known to be a calcium channel blocker, inhibiting calcium influxes into the cell^34,35^. Taken together, our data suggest the *Lr14a*-mediated resistance is related to changes of calcium fluxes.

Our results demonstrate the involvement of LR14A, a representative of an ANK-TM protein family, in race-specific disease resistance in wheat. Future studies on LR14A and ACD6 will show if these proteins act as interactors with PRRs, helper NLRs^36^ or possibly as calcium channels at the plasma membrane. The absence of molecular diversity in the *Lr14a* gene in wild emmer wheat, the domesticated durum landraces and in hexaploid wheat suggests a low selection pressure for diversification of *Lr14a*, possibly because of an indirect recognition of AvrLR14A. Given the race-specific induction of *Lr14a* by an avirulent *P. triticina* isolate, it is tempting to speculate that AvrLR14A could be a transcription factor binding to the *Lr14a* promoter similar to transcription activator-like effector nucleases (TALEN)^37,38^ in bacteria of the genus *Xanthomonas*. However, LR14A might also be the direct target of AvrLR14A with the absence of diversity caused by a short evolutionary time since the introgression of the gene into the wheat gene pool. Specifically, the ANK domain could be a direct target of pathogen effectors. Recently, the wheat stripe rust resistance gene *YrU1* was found to encode an NLR protein with an integrated ANK domain^39^. The ANK domain of *YrU1* is derived from ANK-TM proteins and possibly serves as a decoy for pathogen effectors (Supplementary Fig. 6d). Together with our results, this suggests that multiple ANK-TM proteins might be involved in race-specific disease resistance in wheat, which forms the basis to exploit this protein family in disease resistance breeding.

## Methods

### Plant materials

The bread wheat (*Triticum aestivum*) cultivars (cv.) Arina and Forno (Swiss winter wheats), and NILs (near isogenic lines) Arina*LrFor* (BC2F(5-12)-85), Arina*Lr14a* (BC3F9-85-2019)^4^ were used to clone *Lr14a* (for primers and PCR conditions see Supplementary Data 9). A subset of 158 lines of a RIL (recombinant inbred lines) population (cv. Arina X cv. Forno)^2^ was genotyped for *Lr14a* (for primers and PCR conditions see Supplementary Data 9) and phenotyped after infection with leaf rust. A worldwide collection of 99 wheat and nine spelt (*T. aestivum subspecies spelta*) accessions, available in our lab were genotyped for *Lr14a* presence and allelic diversity as well as for infection type. In addition, 43 accessions from the Whealbi collection^19^, 11 lines from a previous study^40^ of our lab, and 65 wild tetraploid Emmer genotypes (*Triticum dicoccoides*) from another study^17^ of our lab were genotyped for *Lr14a* presence and allelic diversity as well as partially for infection type. A Turkish collection kindly provided by Hakan Ozkan, consisting of 201 wild emmer and 71 *Triticum durum* cultivars and landraces available in his lab were also tested with the *Lr14a* marker. All these lines are listed in Supplementary Data 1 and 2.

### EMS mutagenesis

EMS mutagenesis of Arina*LrFor* (BC2F5-85) and (BC2F10-85) was performed with a concentration of 0.6% and 0.45% EMS (Sigma Aldrich, St. Louis, Missouri, USA), respectively. Seeds were incubated 16 h in water at 4 °C, dried for 8 h on filter paper, and incubated for 16 h with shaking at room temperature in EMS solution. After washing three times for 30, 45, and 60 min, respectively, and for another 30 min under running tap water, seeds were pre-germinated on humid filter paper. Three thousand seeds of BC2F5-85 were mutagenized and pre-germinated seeds were propagated in the field. Single spikes of M0 plants were harvested and M1 plants were infected in the field with leaf rust. The selected susceptible candidate mutant lines were analyzed under field infection conditions over four generations. Three mutant lines were repeatedly phenotyped as susceptible and later confirmed to be susceptible at the seedling stage (BC2F5-85-M5-167, BC2F5-85-M5-983, BC2F5-85-M5-2659). Three thousand seeds of BC2F10-85 were mutagenized and seedling stage infections of the M0 progeny (isolated single spike) revealed four independent susceptible mutant lines (BC2F10-85-M1-7, BC2F10-85-M1-11, BC2F10-85-M1-67, BC2F10-85-M1-71), from which BC2F10-85-M1-11 could not be propagated to the next generation.

### Leaf rust infection

Leaf rust infections at seedling and adult plant stage were performed as described^4^, with *Puccinia triticina* isolates of a Swiss collection^41^ available in our lab. Briefly, infections at the seedling stage were performed with leaf rust spores stored in tubes in -80 °C. They were thawed at 42 °C for 1 min, mixed with FC-43 oil (3 M™ Fluorinert™ FC-43, 3 M Electronics, Zwijndrecht, Belgium), and then sprayed with a high-pressure air sprayer on humid plants, 10 days after sowing at two-leaf stage. Seeds were sown in soil (Rasenerde (20% organic matter, pH (CaCl_2_) 6.5, 1.4 g/L salt content (KCl), filler (DIN EN 12580)), ökohum GmbH, Herrenhof, Switzerland) and watered initially with 2 L water, mixed with fertilizer (Wuxal^®^ Profi (2.5 mL/L), Maag Garden, Syngenta, Düsseldorf, Germany) and growth inhibitor (Cycocel® Extra (1 mL/L), Omya AG, AGRO, Oftringen, Switzerland). After infection, plants dried for 30 min, were then covered with a plastic foil cover to maintain a high level of humidity, and kept in the dark for 24 h. After 9 days of light incubation (60% humidity, 16 h 350 μM light, 20 °C, 8 h dark, 16 °C) under the plastic foil cover, leaf rust infected 2nd leaves where glued on transparent foil and scanned at 720 dots per inch on an Epson Perfection V850 Pro scanner (Epson, Kloten, Switzerland). For scoring, the leaf rust infection phenotypes were evaluated according to the classifications established by McIntosh and colleagues^5^. 1 = small uredia with necrosis, 2 = small to medium sized uredia with necrosis or chlorosis, 3 = medium sized uredia with or without chlorosis, ; = hypersensitive flecks.

Leaf rust infections in the field were performed with a mixture of 16 Swiss *P. triticina* isolates^41^. In spring (March), two weeks old susceptible wheat seedling pots (cv. Walter 60%, cv. Arina 20%, cv. Frisal 20%) were infected with a leaf rust spore/talcum powder mixture and grown for two weeks in a greenhouse (60% humidity, 16 h 350 μM light, 17 °C, 8 h dark, 13 °C). After two weeks of acclimatization, they were transferred next to the infection rows (33% cv. Arina, 33% cv. Bernina, 33% cv. Frisal) which are positioned right and left from three rows of testing plants, 40 seeds/row. Three to four replicates of testing plants were randomly placed. The field was located in Reckenholz, Zurich, Switzerland. Scoring was done as soon as leaf rust uredia were visible on flag leaves (summer, mid/end June, depending on climate conditions) and performed for around two weeks every second day until flag leaves dried out. For scoring, coverage of leaf rust uredia on flag leaves was estimated as percentage^42^ and compared with susceptible and resistant controls.

### Chromosome flow sorting

Wheat cv. Arina and Arina*LrFor* carry the well-described 7BL/5BL translocation^43^. Translocation chromosome 7BL/5BL was purified by flow cytometric sorting^44^. Briefly, suspensions of mitotic metaphase chromosomes were prepared from synchronized wheat seedling root tips^45^. A fluorescein isothiocyanate (FITC) conjugate was used to label GAA microsatellites on chromosomes in suspension by FISHIS^46^ while 4′,6-diamidino-2-phenylindole (DAPI), 2 μg/ml, was used to stain chromosomal DNA. The chromosome samples were analyzed at a rate of 2000 chromosomes/s by a FACSAria SORP (BD Biosciences, San Jose, California, USA) and sort windows were set up on FITC versus DAPI dot plots to sort chromosome 7BL/5BL in wheat. The chromosomes were sorted at rates of 25/s into 0.5-ml PCR tubes containing 40 μl deionized water. For each of the mutants, 25-35 thousand 7BL/5BL chromosomes were sorted, which yielded DNA for three independent multiple displacement amplifications^47^ using the Illustra GenomiPhi V2 DNA amplification kit (GE Healthcare Life Sciences, Pittsburgh, Pennsylvania, USA). To achieve a higher sequence representation, amplified DNA samples derived from each line were pooled. To determine the purity of the 7BL/5BL chromosome in flow-sorted fractions, 2000 chromosomes were sorted into a 5-μl drop of P5 buffer on a microscope slide during each sort run and sorted chromosomes were identified by FISH using probes for GAA microsatellites and Afa family repeat^48^. Purities of the 7BL/5BL chromosome and total DNA yields for the three *Lr14a* mutant lines were as follows: BC2F5-85-M5-167 = 95% purity and 8.29 µg DNA; BC2F5-85-M5-983 = 95% purity and 8.75 µg DNA; BC2F5-85-M5-2659 = 96% purity and 12.07 µg DNA.

### Sequencing

Sanger sequencing was performed in house on an ABI 3730 (Thermo Fisher Scientific, Waltham, Massachusetts, USA). Primers use for sequencing are given in Supplementary Data 9. Illumina sequencing library preparation and Illumina sequencing were performed by the Functional Genomics Center Zurich, (https://fgcz.ch/, Zurich, Switzerland). Enriched chromosome 7BL/5BL with a size of 977 Mb^9^ from three Arina*LrFor* EMS mutant lines were sequenced on an Illumina HiSeq 2500 (Illumina, San Diego, California, USA) to an average coverage of 20-fold. Whole-genome-de-novo paired-end sequencing of 150 bp DNA fragments was performed.

### MutChromSeq with synthetic assembly and synthetic Illumina reads

The flow-sorted chromosome 7B EMS mutant Illumina raw reads were analyzed for their quality using FastQC (http://www.bioinformatics.bbsrc.ac.uk/projects/fastqc). For sequencing adapter removal and quality trimming, cutadapt^49^ and sickle (https://github.com/najoshi/sickle), with the sickle parameter –q = 25 and -l = 20, were used. Arina*LrFor* pseudomolecule version 3 7BL (positions 1–422 Mb) as established as part of the recently published 10+ Wheat Genomes Project^9^ (www.10wheatgenomes.com, https://wheat.ipk-gatersleben.de//) was used for fragmentation into synthetic assemblies with synthetic contig sizes of 3000, 5000, and 7000 bp respectively for three different MutChromSeq runs using the perl command: ‘perl -ne ‘BEGIN{$/ = “ > “}{s/(.*)//;$n = $1;s/\n//g;$i = 0;s/(.{1,${contig_size}})/printf(“>%s_%05d\n%s\n”,$n,++$i,$1);/ge;}’ chromosome7BL.fasta > chromosome7BL_${contig_size}.fasta’. Transposable elements were masked in synthetic assemblies using the transposable element database version 2018 (https://www.botinst.uzh.ch/en/research/genetics/thomasWicker/trep-db.html, http://botserv2.uzh.ch/kelldata/trep-db/index.html).

As reference, synthetic Illumina reads of 150 bp each, paired-end with an insert size of 2,000 bp, without mutations and a coverage of 20-fold were synthesized by using the chromosome 7BL pseudomolecule and the dwgsim program (https://github.com/nh13/DWGSIM) with the command: ‘dwgsim -e 0 -E 0 -d 2000 -N 2 -C 20 -1 150 -2 150 -r 0 -F 0 -R 0 -X 0 -I 1 -y 0 -n 150 -c 0 -S 2 -q G chromosome7BL.fasta reads_chromosome7BL’.

MutChromSeq was performed three times with synthetic Illumina reads as reference wild-type reads and with synthetic assemblies of 3000, 5000, and 7000 bp synthetic contig length. Commands were used according to protocol (https://github.com/steuernb/MutChromSeq)^10^. Adjustments were made in Pileup2XML command (pileupfloat = 0.1, pileupcoverage = 10) and MutChromSeq command (mutchromseqfloat = 0, mutchromseqcoverage = 8, n = 3, z = 1).

Three different MutChromSeq runs resulted in 25 (3000 bp), 26 (5000 bp), and 30 (7000) synthetic candidate contigs (Supplementary Data 10,11,12) The 25 synthetic candidate contigs of the 3000 bp synthetic assembly were filtered for standard EMS mutations (G/C to A/T) by eye. The remaining three synthetic candidate contigs of the 3000 bp MutChromSeq run (Supplementary Data 13) were blasted for gene content with the Chinese Spring annotation: IWGSC Reference Sequence v1.0 Annotation (https://wheat-urgi.versailles.inra.fr/Seq-Repository/Annotations) and checked if EMS mutations lead to coding sequence changes in genes. Two of the remaining three synthetic contigs had only short overlaps with protein-coding genes (38 bp and 209 bp, respectively), while the third one had a hit of 900 bp for a protein-coding gene and also the EMS mutations occurred within the coding sequence.

### Virus-induced gene silencing

For virus-induced gene silencing (VIGS) target design, exon 1 and exon 3 of *Lr14a* were aligned respectively against the recently published Arina*LrFor* genome sequence^9^ using BLASTN to find the sequences with the lowest similarity to the rest of the genome. Based on that, two regions with lowest similarities (exon 1, 78-83% similarity, 213 – 486 bp = VIGS1; exon 3, 84% similarity, 20 – 287 bp = VIGS3) were selected as VIGS target sequences.

VIGS cloning and infection were performed as described^50–53^, using a phytoene desaturase gene silencing construct to induce photo bleaching, as positive control. For cloning the VIGS1 or VIGS3 amplicon (primers used are listed in Supplementary Data 9) into the vector pBS-BSMV-γ, respectively, the restriction sites *Not*I and *Pac*I in antisense direction were used. The virus genome is composed of the pBS-BSMV-α, pBS-BSMV-β, and pBS-BSMV-γ plasmids, where the γ plasmid carried the VIGS target sequence VIGS1 or VIGS3. The wild-type (WT) viral genome was used as control. For in vitro synthesis of virus RNA, the Invitrogen™ mMESSAGE mMACHINE™ T7 Transcription Kit (Thermo Fisher Scientific, Waltham, Massachusetts, USA) was used. For virus infection, seeds in soil were kept at 4 °C for 7 days. Then, seedlings were placed in a growth chamber (60% humidity, 16 h 350 μM light, 23 °C, 8 h dark, 16 °C). Seedlings at this time were at the two-leaf stage and the first leaf was completely inoculated with viral RNA. At day 14 after virus infection, a seedling leaf rust infection was performed and 10 days later, leaf rust phenotypes on the 3rd and/or 4th leaf were documented. Leaf rust uredia and decreased hypersensitive flecks count was done in a 0.5 × 2 cm square in the middle of leaves from one representative experimental repetition (*n* = 8, 7, 6 leaves for VIGS1, VIGS3, WT, respectively).

### Bioinformatics analysis

The programs Dotter, Water, Clustalx, Clustalw, MrBayes, FigTree, and blast were all obtained from Ubuntu repositories (ubuntu.com). Analysis of sequenced PCR products was performed with CLC Main Workbench 20.0.2 and versions below. Ankyrin (ANK) repeats were annotated manually through dot plots of the predicted LR14A protein against itself and by identifying previously described conserved ANK amino acid motifs^12,54^. The three-dimensional structure of the LR14A ANK domain was predicted with RaptorX^55^, (http://raptorx.uchicago.edu/), Phyre2^56^, (http://www.sbg.bio.ic.ac.uk/~phyre2/html/page.cgi?id=index), and HHPred^57^, (https://toolkit.tuebingen.mpg.de/tools/hhpred). Transmembrane domains were predicted with TMHMM server v 2.0 (http://www.cbs.dtu.dk/services/TMHMM/).

### Molecular dating

Regions homologous to the *Lr14a* locus in bread wheat cv. Lancer and Spelt wheat PI190962 were identified by blastn using the coding sequence of *Lr14a* as query. In the case of bread wheat cv. Chinese Spring (which does not contain *Lr14a*), the *Lr14a* region was identified by aligning the homologous regions of chromosome 7B in the two cultivars. The regions containing the gene and 200 kb of flanking sequence were extracted from the pseudomolecule sequences and aligned with Dotter in order to determine the extent of the alignable sequences. Once boundaries of conserved sequences were established, homologous regions were aligned with the emboss program Water (https://www.ebi.ac.uk/Tools/psa/emboss_water/). For these alignments, only intergenic sequences (sequences >500 bp up- or downstream from annotated genes) were used. Divergence time calculations between haplotypes were performed using a substitution rate of 1.3 E-8 substitutions per site per year^58,59^. We only used nucleotide substitutions for molecular dating, and insertions and deletions were ignored^58^.

### Collinearity breakpoint between the *Lr14a* locus in Arina*LrFor* and Chinese Spring

The *Lr14a* locus is fragmented to different degrees in the genome assemblies of bread wheat cultivars Arina*LrFor*, Lancer, Chinese Spring, and spelt wheat accession PI190962. Because comparison of Chinese Spring and Arina*LrFor* was central to our model for the evolution of the *Lr14a* locus, the breakpoints of sequence collinearity between Chinese Spring and Arina*LrFor* were confirmed by PCR. Here, the precise end points of sequence collinearity were determined with the program Dotter. Subsequently, primer pairs (primers are listed in Supplementary Data 9) were generated to bridge the breakpoints in Arina*LrFor*. Sequenced PCR fragments aligned to the genomic sequences could confirm the integrity of the sequences in the breakpoint regions.

### Phylogenetic analysis

Proteins with the ANK-TM domain organization form a very large family with dozens of members in all angiosperm genomes. To reduce complexity and shorten computation time, three types of LR14A homologs were selected as follows: 1. the top ten hits of a blastp search of LR14A against Arabidopsis proteins, 2. the top ten hits of a blastp search of LR14A against all proteins of the wheat (cv. Chinese Spring) B genome, and 3. the top ten hits of a blastp search of Arabidopsis ACD6 against all proteins of the wheat (cv. Chinese Spring) B genome. The last search was performed to identify the closest homologs of ACD6 in wheat. The wheat dataset was reduced to the B genome to simplify the tree and shorten computation time. ANK-TM-type proteins that were previously described to be involved in plant stress response were added to the selection. These included ACD6^19,24,60^, BDA1^21^, and ITN1^20^ from Arabidopsis and ZmACD6^22^ from maize. Protein sequences were aligned with Clustalw (obtained from ubuntu.com repositories) using a gap opening penalty of 5 and a gap extension penalty of 0.01. The multiple alignments were converted to nexus format with Clustalx (obtained from ubuntu.com repositories). A phylogenetic tree for LR14A homologs was constructed with MrBayes^61^, using parameters lset = 6 and rates = invgamma and by running Markov chain Monte Carlo simulation (mcmc) for 700,000 generations with a sample frequency of 10. Trees were summarized using a burn-in of 25%. The consensus tree was visualized with FigTree (http://tree.bio.ed.ac.uk/software/figtree/). The phylogenetic analysis of the ANK domains of the proteins was done in the same way as for the full-length proteins described above. The difference was that the ANK domain of YrU1^39^ was added to the datasets and that the mcmc simulation of MrBayes was run for 1,000,000 generations. The length of the ANK domain used for the analysis was determined by aligning YrU1 with LR14A homologs.

### LR14A localization

The coding sequence of *Lr14a* was cloned (primers are listed in Supplementary Data 9) with the Invitrogen™ pENTR™ D-Topo® Cloning Kit and Invitrogen™ Gateway® Lr Clonase™ II Plus Enzyme Mix (Thermo Fisher Scientific, Waltham, Massachusetts, USA) into the pGWB506 vector (pGWB506 was a gift from Tsuyoshi Nakagawa (Addgene plasmid # 74848; http://n2t.net/addgene:74848; RRID:Addgene_74848)^62^) to attach an N-terminal enhanced green fluorescence protein (eGFP) to LR14A, expressed by a cauliflower mosaic virus (CaMV) 35 S promotor. As plasma membrane marker, an N-terminal monomeric red fluorescence protein (mRFP) tagged *AtREM1.2* (At3g61260) coding sequence under a CaMV 35 S promotor was used^63^. Live cell imaging was performed using a Leica SP5 confocal laser scanning microscopy system (Leica, Wetzlar, Germany) equipped with Argon, DPSS, and He-Ne lasers and hybrid detectors and the LAS AF Version 2.7.3.9723 Leica Microsystems CMS GmbH Software. Imaging was performed as previously described^64^, with minor modifications. Briefly, *Nicotiana benthamiana* transformation, with *Agrobacterium tumefaciens* (strain GV3101), carrying the plasmids of interest as transformation vector, was performed as described^65^. Three days after *N. benthamiana* infiltration, leaf samples of 5 × 5 mm were transferred between a glass slide and a cover slip in a drop of water. For plasmolysis induction, samples were incubated 10–15 min in 4% NaCl prior to imaging. Fluorescence was observed with excitation wavelengths of 488 nm and emission wavelengths of 490 to 550 nm for eGFP and with excitation wavelengths of 561 nm and emission wavelengths of 580–640 nm for mRFP. Chlorophyll autofluorescence was captured using emission wavelengths of 700–720 nm after excitation of the samples at 488 nm. Fluorescence intensities across plasma membrane sections were measured using the plot line plugin in Fiji software (https://fiji.sc/). Experiments were performed using strictly identical confocal acquisition parameters (e.g. laser power, gain, zoom factor, resolution, and emission wavelengths reception), with detector settings optimized for low background and no pixel saturation. Pseudo-colored images were obtained using “Green”, “Magenta”, and “Turquoise” look-up-table (LUT) of Fiji software.

### RNAseq experiments and analysis

RNA was extracted with the SV Total RNA Isolation System (Promega, Madison, Wisconsin, USA). *N. benthamiana* leaves were transiently transformed with *A. tumefaciens* (strain GV3101), carrying the plasmids of interest as described^65^. Leaves were transiently transformed with either a human influenza hemagglutinin (HA) N-terminal tagged *Lr14a* coding sequence carrying vector pIPKb004^66^, or an empty vector, both co-infiltrated with *A. tumefaciens* carrying a P19 vector (P19 protein from tomato bushy virus (TBSV)^67^) sampled 27 hpi (hours post-inoculation). Wheat leaves inoculated with *P. triticina* isolates 96209 (*Lr14a* avirulent isolate), 95037 (*Lr14a* virulent isolate) or infection medium, respectively, were sampled 8 days post-inoculation. *N. benthamiana* RNA samples were sequenced on a Novaseq PE150 (Illumina, San Diego, California, USA) to a total data amount of 6 Gbp/20 M reads per sample. Wheat samples were sequenced on a Novaseq 6000 S4 (Illumina, San Diego, California, USA) to a total data amount of 12 Gbp/40 M reads per sample.

In order to quantify the transcriptomic response induced by the transient expression of *Lr14a* in *N. benthamiana*, we used the RNA-Seq analysis pipeline Salmon^68^ with standard parameters. RNAseq libraries of *N. benthamiana* plants in the presence or absence of *Lr14a* at 27 h post-infiltration were quantified in mapping-based mode on the *N. benthamiana* coding sequence annotation (version 1.0.1). The estimated number of mapped reads were further considered for differential expression (DE) analysis. We used the R package edgeR^69^ and only the genes with a log2FC > |1.5| and an adjusted *p*-value (FDR) < 0.05 were considered as differentially expressed^70^. To further investigate the interactions between *N. benthamiana* genes that were up-regulated by the transient expression of *Lr14a*, we performed a gene enrichment analyses against the Gene Ontology (GO) database. GO terms enriched in up-regulated genes were identified using the R package GOseq^71^, and the results with a corrected *p*-value smaller than 0.05 were considered significant. AgriGO v2.0^72^ was finally used to generate the directed acyclic graph (DAG) of the GO molecular functions. The same workflow was used for analyzing the wheat transcriptomic data. Coding sequences and corresponding GO identifiers, blast homologs and gene functions, were retrieved from the International Wheat Genome Sequencing Consortium Reference Sequence (IWGSC RefSeq) v1.0 annotation.

Finally, the wheat transcriptomic response induced by LR14A was compared with other RNA-Seq studies in which wheat plants were challenged with different abiotic and biotic stresses. For this purpose, we only considered the DEGs that were identified between Thatcher*Lr14a* and Thatcher, upon infection of the avirulent *P. triticina* isolate at 8 dpi. The transcriptomic data were downloaded from wheat-expression.com^70,73,74^ and the following stress treatments were considered: *Zymoseptoria tritici* (Zt; ERP009837), *Fusarium graminearum* (Fg; ERP013829), stripe rust and wheat powdery mildew (Sr, Pm; ERP013983, SRP041017), *Fusarium pseudograminearum* (Fp; SRP048912), PAMPs (chitin and flg22) and cold (SRP043554). Differential gene expression analysis was performed as mentioned above and the R package pheatmap was used for comparing the log2FC values of the LR14A-induced DEGs across all the different stress treatments. In order to improve the data visualization, log2FC values were scaled to Z-scores with the argument scale = ‘row’.

### *Lr14a* phenotyping and LaCl_3_ phenotype inhibition on *Nicotiana benthamiana* lower leaf surface

*A. tumefaciens* carrying an overexpression construct for wild-type (WT) *Lr14a* coding sequence in the vector pIPKb004^66^, EMS loss of function mutant (L362F) *Lr14a* coding sequence in the vector pIPKb004^66^, N-terminal enhanced green fluorescence protein (eGFP) tagged *Lr14a* coding sequence in a pGWB506 vector (pGWB506 was a gift from Tsuyoshi Nakagawa (Addgene plasmid # 74848; http://n2t.net/addgene:74848; RRID:Addgene_74848)^62^) and N-terminal human influenza hemagglutinin (HA) tagged *Lr14a* coding sequence in the vector pIPKb004^66^ was used to transiently transform *N. benthamiana* as described^65^. Lanthanum (III) chloride (LaCl_3_) was infiltrated 1–2 h post Agrobacteria infiltration in a concentration of 2 mM, dissolved in water. Water was used for infiltration on control leaf spots. Leaves were scanned 2 or 3 days post-inoculation on an Epson Perfection V850 Pro scanner (Epson, Kloten, Switzerland), or under a Fusion FX Imaging System (Vilber Lourmat, Eberhardzell, Germany) as described for hypersensitive response (HR) measurements^65^.

### Salicylic acid extractions and measurements

Salicylic acid extractions and measurements were performed as previously described^75^. Briefly, Arina*LrFor* WT and mutant plants were infected 10 days after germination with the avirulent *P. triticina* isolate 96209 and leaf samples were taken 8 dpi. SA quantification was performed using ultra-high performance liquid chromatography-tandem mass spectrometry (UHPLC-MS/MS). Fresh frozen tissues were ground in liquid nitrogen and approximately 100 mg of powder was weighed in a microcentrifuge tube. To this tube 990 µL of ethylacetate:formic acid (99.5:0.5, v/v), 1 ng of SA-d4 and 3–5 glass beads were added, and the tube was shaken at 30 Hz for 3 min in a mixer mill. After centrifugation, the supernatant was reserved and the pellet re-extracted with 0.5 mL of extraction solvent. The supernatants were combined, evaporated and the dry residue was reconstituted in 100 µL of methanol 70%. A 5 µL injection was made in the UHPLC-MS/MS system which was composed of a Dionex Ultimate 3000 RSLC (Thermo Fisher Scientific, Waltham, Massachusetts, USA) coupled to a 4000 QTRAP (AB Sciex, Framingham, Massachusetts, USA). A gradient separation was performed on a 50 × 2.1 mm Acquity UPLC BEH C18 column (Waters, Milford, Massachusetts, USA) using mobile phases of water and acetonitrile, both supplemented with 0.05% formic acid. The mass spectrometer was set in negative electrospray ionization and the transitions 137/93 and 141/97 were monitored for SA and SA-d4, respectively.

### Molecular methods to map, clone, and characterize *Lr14a*

Genomic wheat DNA was extracted following the Cetyltrimethylammonium bromide (CTAB) extraction protocol^76^, using cold dichlormethane:isoamylalcohol (24:1) instead of phenol:chloroform:isoamylalcohol (48:48:4). Genotyping for *Lr14a* was performed using primers and PCR conditions listed in Supplementary Data 9. PCR amplification of the *Lr14a* gene (for primers and conditions see Supplementary Data 9) had to be performed in two fragments, as microsatellite repeats in the first intron blocked polymerase activity and a full-length amplification of the *Lr14a* gene was not possible. For mRNA extraction of infected 2nd leaves, the Invitrogen™ Dynabeads™ mRNA DIRECT™ Kit (Thermo Fisher Scientific, Waltham, Massachusetts, USA) was used. The mRNA integrity was validated by gel electrophoresis as well as spectrophotometric analysis using a NanoDrop 1000 Spectrophotometer (Thermo Fisher Scientific, Waltham, Massachusetts, USA). The DNA Ladder used was GeneRULER™ 1 kb Plus DNA Ladder (Thermo Fisher Scientific, Waltham, Massachusetts, USA). The mRNA was used to synthesize cDNA with the iScript Advanced cDNA Synthesis Kit for RT-qPCR (BIO-RAD, Hercules, California, USA). Sequencing of cDNA of *Lr14a* (primers are listed in Supplementary Data 9), extracted from leaf rust infected leaves revealed a single cDNA version. Therefore, we concluded that no alternative splicing variants are produced. For race PCR (primers are listed in Supplementary Data 9) the SMARTer^®^ RACE 5′/3′ Kit (Takara Bio Europe, Saint-Germain-en-Laye, France) was used. Amplification of the 5′ and 3′ untranslated region (UTR) revealed no variations in UTR length. Generated cDNA was also used for qPCR (primers are listed in Supplementary Data 9) using the KAPA SYBR® FAST qPCR Master Mix (2X) Kit (Sigma Aldrich, St. Louis, Missouri, USA). Three technical replicates each of three biological replicates were used for qPCR. In addition, pathogenesis response marker genes (*PR1, 2, 5*)^15^ (primers are listed in Supplementary Data 9) were used for qPCR characterization of Arina*LrFor* WT and mutant lines.

### Reporting summary

Further information on research design is available in the Nature Research Reporting Summary linked to this article.

## Supplementary information

Supplementary Information

Reporting Summary

Description of Additional Supplementary Files

Supplementary Data 1

Supplementary Data 2

Supplementary Data 3

Supplementary Data 4

Supplementary Data 5

Supplementary Data 6

Supplementary Data 7

Supplementary Data 8

Supplementary Data 9

Supplementary Data 10

Supplementary Data 11

Supplementary Data 12

Supplementary Data 13

## Data Availability

All data supporting the findings of this work are available within the paper and its supplementary information files. A reporting summary for this article is available as a supplementary information file. The datasets and plant materials generated and analyzed during the current study are available from the corresponding author upon request. Sequence data were deposited at the NCBI GenBank under the accession number MT123593 (*Lr14a* coding sequence), and at the NCBI sequence read archive (SRA) database under the accession number PRJNA529355 (flow-sorted chromosome 7B of three Arina*LrFor Lr14a* EMS mutants) or are available via the recently published 10+ Wheat Genome Project^9^ [www.10wheatgenomes.com or https://wheat.ipk-gatersleben.de//]. RNAseq raw data were deposited at the NCBI SRA database under the accession number PRJNA674985 for *Triticum aestivum* or PRJNA674843 for *Nicotiana benthamiana*. Source data are provided with this paper.

## Source data

Source Data
