# Supplementary material for: A membrane-bound ankyrin repeat protein confers race-specific leaf rust disease resistance in wheat
Source: Nat Commun. 2021 Feb 11;12:956. doi: 10.1038/s41467-020-20777-x (PMC7878491; doi:10.1038/s41467-020-20777-x)
Supplement: Supplementary file 1 — Supplementary Information [file 41467_2020_20777_MOESM1_ESM.pdf]

A membrane-bound ankyrin repeat protein confers race-specific leaf rust disease resistance in wheat

Kolodziej *et al.*

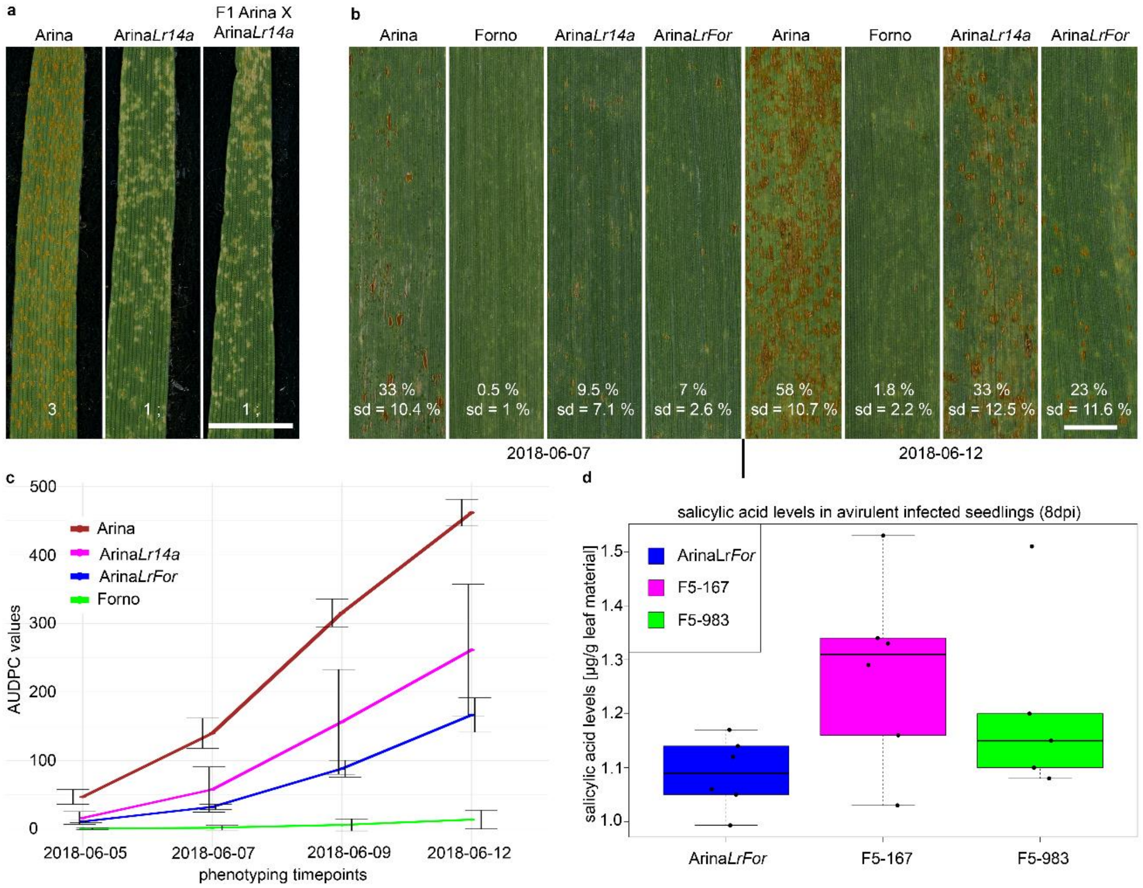

Supplementary Fig. 1. Genetic and phenotypic analysis of *Lr14a* containing wheat lines. **a,** Dominant gene action of *Lr14a*. Arina, Arina*Lr14a* and an F_1 seedling derived from a cross between both lines were infected with the *Lr14a* avirulent *Puccinia triticina* isolate 96209 ten days after sowing. Pictures show second leaves ten days post inoculation (dpi). Arina was susceptible while Arina*Lr14a* and F_1 seedling showed resistance. Scale bar = 0.5 cm **b,** Field infection with a mixture of 16 Swiss *P. triticina* isolates virulent and avirulent on *Lr14a*[1]. The average flag leaf coverage by leaf rust (Arina n = 13, Forno n = 4, Arina*Lr14a* n = 4, Arina*LrFor* n = 13) is indicated for two different time points. For scoring of adult plant plots, coverage of leaf rust uredia on flag leaves were estimated in percentage[2]. sd = standard deviation, scale bar = 1 cm. **c,** Area under disease pressure curve (AUDPC) of the field scoring values 2018 over the complete scoring time. Arina was highly susceptible while Forno was highly resistant. Arina*LrFor* showed strong resistance while Arina*Lr14a* showed moderate resistance. n = 4 biological replicates. Error bars represent the standard deviation of the presented mean. **d,** Salicylic acid (SA) levels (ng/g plant material) measured in 2nd leaves. Plants were infected ten days after sowing and sampled 8 dpi with the avirulent *P. triticina* isolate 96209. Arina*LrFor* (n = 6) showed no significant differences in SA levels compared to the two *Lr14a* mutants F5-167 (n = 6) and F5-983 (n = 5). Statistics: A Levene test, anova and Tukey-HSD were performed. For boxplots: minimum value = lower whisker, maximum value = upper whisker, median = middle value of box, lower quartile = median of lower half of dataset, upper quartile = median of upper half of dataset, datapoint outside of whiskers = potential outlier. Source data are provided as a Source Data file.

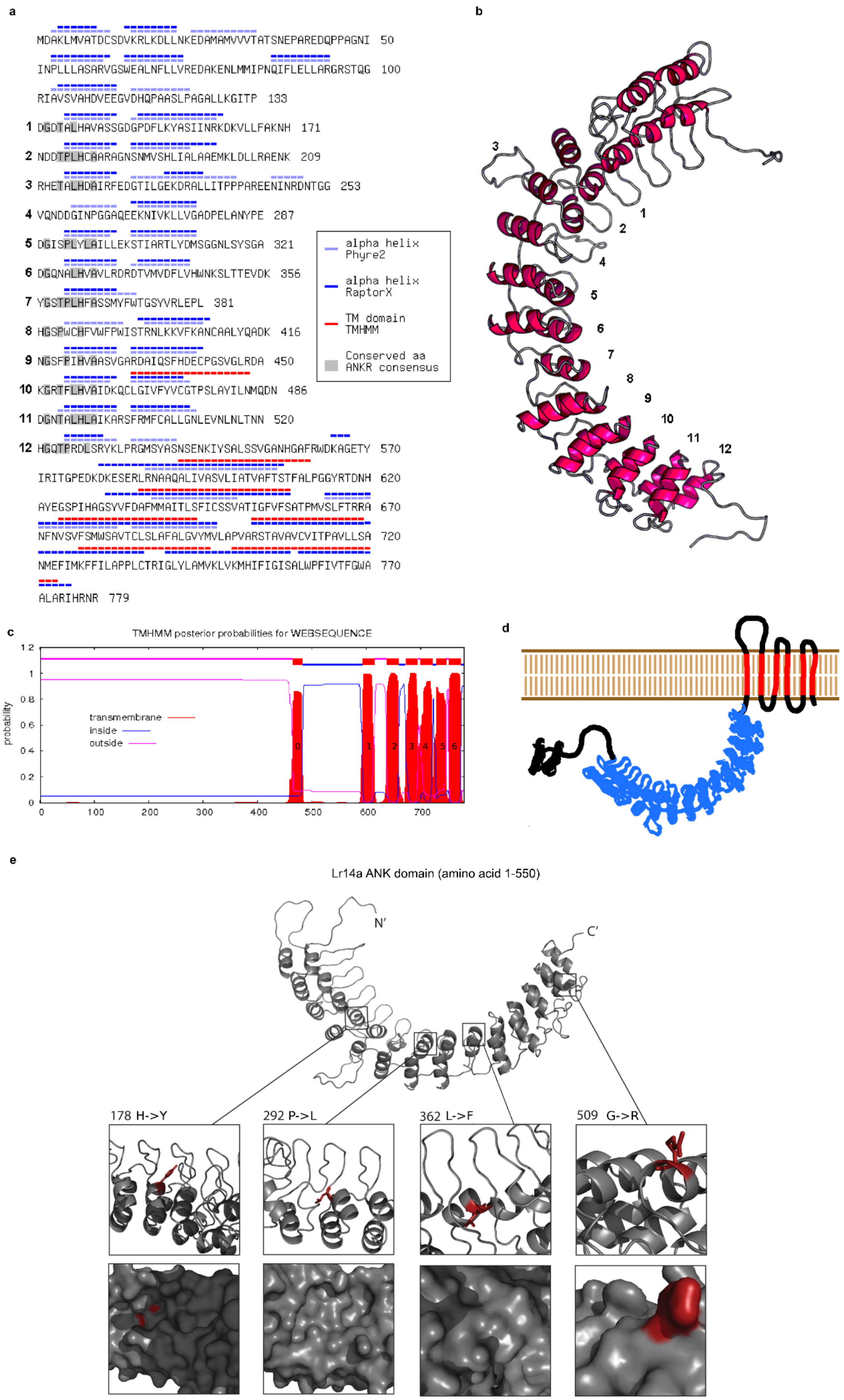
a
MDAKLMVATDCSDVKRLKDLLNKEDAMAMVVVTATSNEPAREDQPPAGNI 50
INPLLLASARVGSWEALNFLLVREDAKENLMMIPNQIFLELLARGRSTQG 100
RIAVSVAHDVEEGVDHQPAASLPAGALLKGITP 133
1 DGDTALHAVASSGDGPDFLKYASIINRKDKVLLFAKNH 171
2 NDDTPLHCAARAGNSNMVSHLIALAAEMKLDLLRAENK 209
3 RHETALHDAIRFEDGTILGEKDRALLITPPPAREENINRDNTGG 253
4 VQNDDGINPGGAQEEKNIVKLLVGADPELANYPE 287
5 DGISPLYLAILLEKSTIARTLYDMSGGNLSYSGA 321
6 DGQNALHVAVLRDRDTVMVDFLVHWNKSLTTEVDK 356
7 YGSTPLHFASSMYFWTGSYVRLEPL 381
8 HGSPWCHFVWFPWISTRNLKKVFKANCAALYQADK 416
9 NGSFPIHVAASVGARDAIQSFHDECPGSVGLRDA 450
10 KGRTFLHVAIDKQCLGIVFYVCGTPSLAYILNMQDN 486
11 DGNTALHLAIKARSFRMFCALLGNLEVNLNLTNN 520
12 HGQTPRDLSRYKLPRGMSYASNSENKIYSALSSVGANHGAFRWDKAGETY 570
IRITGPEDKDKESERLRNAAQALIVASVLIATVAFTSTFALPGGYRTDNH 620
AYEGSPIHAGSYVFDAFMMAITLSFICSSVATIGFVFSATPMVSLFTRRA 670
NFNVSVFSMWSAVTCLSLAFALGVYMVLAPVARSTAVAVCVITPAVLLSA 720
NMEFIMKFFILAPPLCTRIGLYLAMVKLVKMHIFIGISALWPFIVTFGWA 770
ALARIHRNR 779
alpha helix Phyre2
alpha helix RaptorX
TM domain TMHMM
Conserved aa ANKR consensus
b
1 2 3 4 5 6 7 8 9 10 11 12
c
TMHMM posterior probabilities for WEBSEQUENCE
probability
transmembrane
inside
outside
0 1 2 3 4 5 6
0 100 200 300 400 500 600 700
0 0.2 0.4 0.6 0.8 1 1.2
d
e
Lr14a ANK domain (amino acid 1-550)
N'
C'
178 H->Y
292 P->L
362 L->F
509 G->R

Supplementary Fig. 2. Analysis of the LR14A protein sequence. **a,** Annotated protein sequence. Amino acids that are predicted to be part of α-helices are marked with horizontal lines in light blue (Phyre2 prediction) and dark blue (RaptorX prediction). Amino acids predicted by TMHMM to be part of transmembrane (TM) domains are marked with red horizontal lines. Predicted ankyrin (ANK) repeats are numbered 1-12 and aligned according to the previously described ANK repeat consensus sequence[3]. Residues corresponding to the published consensus sequence are shaded in gray. Most ANK repeats consist of two α-helices with the exception of repeats 7 and 12 which are shorter. The Arabidopsis homolog ACD6 has only 9 predicted ANK repeats and 5 transmembrane domains[4,5]. **b,** RaptorX predicted 3-dimensional protein structure of the ANK domain of Lr14a. The prediction includes ten complete ANK repeats, two partial repeats (7, 12), and 12 α-helix pairs (plus two single α-helices 7 and 12). **c,** Prediction of TM domains in LR14A with the TMHMM software v. 2.0 (http://www.cbs.dtu.dk/services/TMHMM/). Note that TM domain 0 is located within the predicted ANK domain and was thus not considered real. We propose the first part of the protein to be intracellular. **d,** Schematic model of the LR14A protein. We propose that the protein is anchored to the plasma membrane with its 6 TM domains (red) while the N-terminal ANK domain (blue) is cytoplasmic. The 6 to 8 (depending on the modeling software) N-terminal α-helices not belonging to the ANK domain are displayed separate (black). **e**, Location of EMS induced amino acid polymorphisms in the ANK repeat domain of the LR14A protein. All amino acid changes occurred in the predicted α-helices that form the structural backbone of the ANK repeats. Note that the mutation L362F was found in two independent EMS mutants.

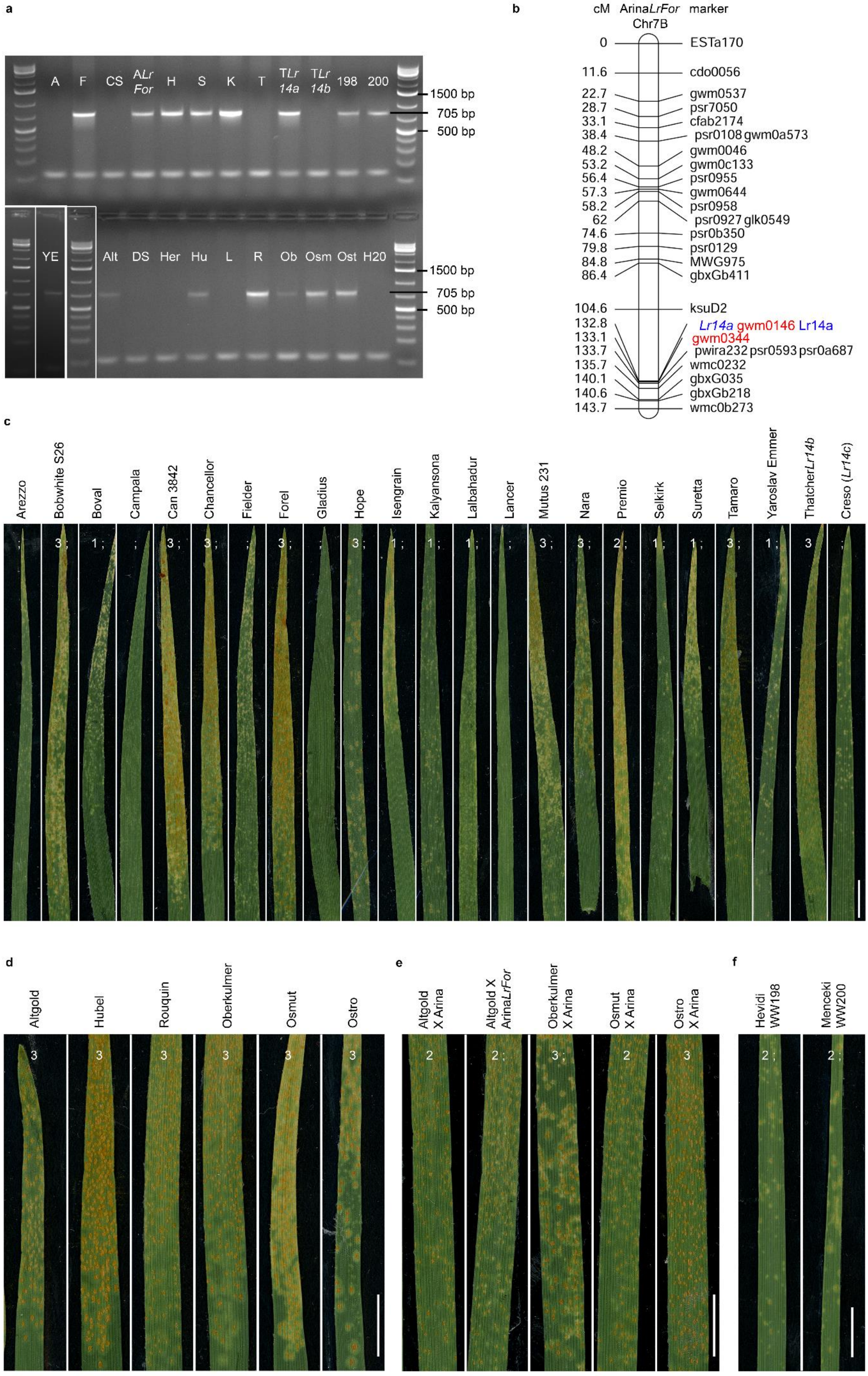
a
A F CS ALr For H S K T TLr 14a TLr 14b 198 200
1500 bp
705 bp
500 bp
YE
Alt DS Her Hu L R Ob Osm Ost H20
1500 bp
705 bp
500 bp
b
cM
ArinaLrFor Chr7B
marker
0 ESTa170
11.6 cdo0056
22.7 gwm0537
28.7 psr7050
33.1 cfab2174
38.4 psr0108 gwm0a573
48.2 gwm0046
53.2 gwm0c133
56.4 psr0955
57.3 gwm0644
58.2 psr0958
62 psr0927 glk0549
74.6 psr0b350
79.8 psr0129
84.8 MWG975
86.4 gbxGb411
104.6 ksuD2
132.8 Lr14a gwm0146 Lr14a
133.1 gwm0344
133.7 pwira232 psr0593 psr0a687
135.7 wmc0232
140.1 gbxG035
140.6 gbxGb218
143.7 wmc0b273
c
Arezzo
Bobwhite S26
Boval
Campala
Can 3842
Chancellor
Fielder
Forel
Gladius
Hope
Isengrain
Kalyansona
Lalbahadur
Lancer
Mutus 231
Nara
Premio
Selkirk
Suretta
Tamaro
Yaroslav Emmer
ThatcherLr14b
Creso (Lr14c)
; 3 ; 1 ; ; 3 ; 3 ; ; 3 ; ; 3 ; 1 ; 1 ; 1 ; ; 3 ; 3 ; 2 ; 1 ; 1 ; 3 ; 1 ; 3 ;
d
Altgold
Hubel
Rouquin
Oberkulmer
Osmut
Ostro
3 3 3 3 3 3
e
Altgold X Arina
Altgold X ArinaLrFor
Oberkulmer X Arina
Osmut X Arina
Ostro X Arina
2 2 ; 3 ; 2 3
f
Hevidi WW198
Menceki WW200
2 ; 2 ;

Supplementary Fig. 3. *Lr14a* marker analysis, mapping, and phenotypic variation. a, Presence of *Lr14a* in selected wheat genotypes determined by a gene-specific, presence/absence PCR marker. The *Lr14a* gene was present in Thatcher*Lr14a* (T*Lr14a*), Arina*LrFor* (A*LrFor*), Forno (F), two durum wheat accessions Hevidi (198) and Menceki (200) as well as the *Lr14a* source stock lines Hope (H), Selkirk (S), and Kalyansona (K) as well as in the original Yaroslav Emmer (YE). Arina (A), Chinese Spring (CS), Thatcher (T), and Thatcher*Lr14b* (T*Lr14b*) do not contain the gene as well as the three spelt cultivars Derenburger Silber (DS), Hercule (Her), and Lueg (L). The other six spelt cultivars, Altgold (Alt), Hubel (Hu), Rouquin (R), Oberkulmer (Ob), Osmut (Osm), and Ostro (Ost) contain the gene (fragment at 705 bp). PCR results were reproduced for each line at least two times. **b,** Genetic map of the *Lr14a* gene specific marker and the *Lr14a* resistance phenotype in a RIL population consisting of 158 genotypes[6]. The red markers gwm0146 and gwm0344 are the closest markers associated with *Lr14a*[7]. The gene-specific marker (Lr14a, blue, no italics) and phenotype (*Lr14a*, blue, italics) of *Lr14a* both co-segregated with gwm0146. **c,** Resistance phenotypes of wheat accessions tested with the *Lr14a*-specific marker (Supplementary Data 2). A variety of different qualitative and quantitative resistance phenotypes were observed which is possibly due to modifier genes[8-10]. The *Lr14b* containing Thatcher line[9] showed a higher susceptibility in contrast to Thatcher*Lr14a*. The *Lr14c* containing Creso line[11] showed a strong resistance phenotype, but as marker and sequencing information showed, Creso contains *Lr14a*. Infection was performed ten days after sowing with the *Lr14a* avirulent *Puccinia triticina* isolate 96209, pictures were taken ten days post inoculation. **d,** Spelt lines containing *Lr14a* showed no hypersensitive flecks and infection type 3 (moderately susceptible). *Lr14a* resistance expression might be reduced due to genetic modifiers[8-10,12]. **e,** Crosses of spelt lines with Arina and Arina*LrFor* result in different genetic backgrounds resulting in non-parental infection types particularly well visible on F_1 seedlings derived from crosses with susceptible cultivar Arina. **f,** Durum wheat accessions Hevidi (WW198) and Menceki (WW200) also showed a mesothetic phenotype with hypersensitive flecks in the minority. For all scans, second leaves were infected with avirulent *P. triticina* isolate 96209 ten days after sowing. Leaves were scanned ten days post infection. 1 = small uredia with necrosis, 2 = small to medium sized uredia with necrosis or chlorosis, 3 = medium sized uredia with or without chlorosis, ; = hypersensitive flecks. Scale bar = 1 cm. Source data are provided as a Source Data file.

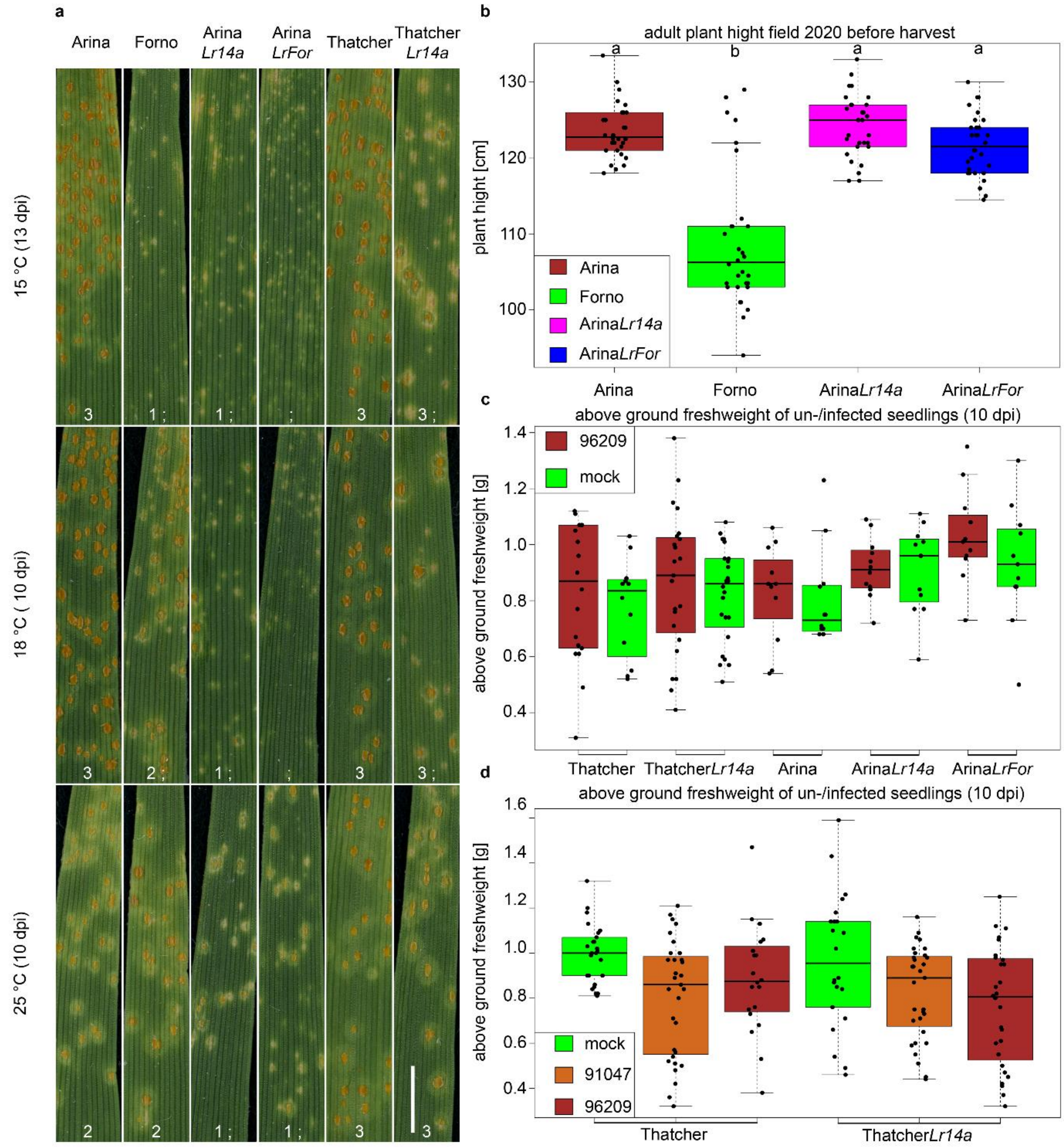

Supplementary Fig. 4. Analysis of *Lr14a* function in wheat at seedling stage. a, Arina and Thatcher, without *Lr14a*, as well as Forno, Arina*Lr14a*, Arina*LrFor* and Thatcher*Lr14a*, with *Lr14a*, were grown at 15, 18 or 25°C after infection. The temperature sensitivity of *Lr14a*[12] is visible as an increased resistance in Forno at 15 °C and high susceptibility of the same line at 25 °C. The other *Lr14a* containing lines showed similar temperature dependence. 1 = small uredia with necrosis, 2 = small to medium sized uredia with necrosis or chlorosis, 3 = medium sized uredia with or without chlorosis, ; = hypersensitive flecks. Scale bar = 0.5 cm. **b,** Arina, Forno, Arina*Lr14a* and Arina*LrFor* plants were analyzed in the field (Zurich, Switzerland) in mature dry state right before harvest. While Forno (with *Lr14a*) was significantly shorter than Arina, the other two *Lr14a*-containing lines in the Arina background were not significantly different from Arina. Plants were infected with a mixture of 16 Swiss *Puccinia triticina* isolates virulent and avirulent on *Lr14a*[1] (see supplementary Fig. 1b, c). n = each line from 3 plots with 10 plants each (30 plants in total per line). Statistics: A Levene test, Anova and Kruskal-Wallis test were performed. **c,** Boxplot showing seedling weights, measuring no significant change in above-ground plant biomass with or without presence of *Lr14a*. *Lr14a* containing (Thatcher*Lr14a* (n = 23/24), Arina*Lr14a* (n = 12/12),

Arina*LrFor* (n = 11/11)) and not containing (Thatcher (n = 18/12), Arina (n = 11/12)) lines infected with an avirulent isolate or non-infected. Statistics: A Levene test, Anova and Tukey-HSD were performed. **d,** Boxplot showing seedling weights, measuring no significant change in above ground plant weight with or without presence of *Lr14a*. Thatcher (n = 25/31/20) and Thatcher*Lr14a* (n = 22/31/28). Statistics: A Levene and Kruskal-Wallis test were performed. For all leaf scans, second leaves were infected with avirulent *P. triticina* isolate 96209, seedlings used for weight determination were infected with avirulent *P. triticina* isolate 96209, virulent *P. triticina* isolate 91047 or treated with infection medium ten days after sowing. Leaves were weighed or scanned ten days post inoculation. For boxplots in (b), (c), (d): minimum value = lower whisker, maximum value = upper whisker, median = middle value of box, lower quartile = median of lower half of dataset, upper quartile = median of upper half of dataset, datapoint outside of whiskers = potential outlier. Source data are provided as a Source Data file.

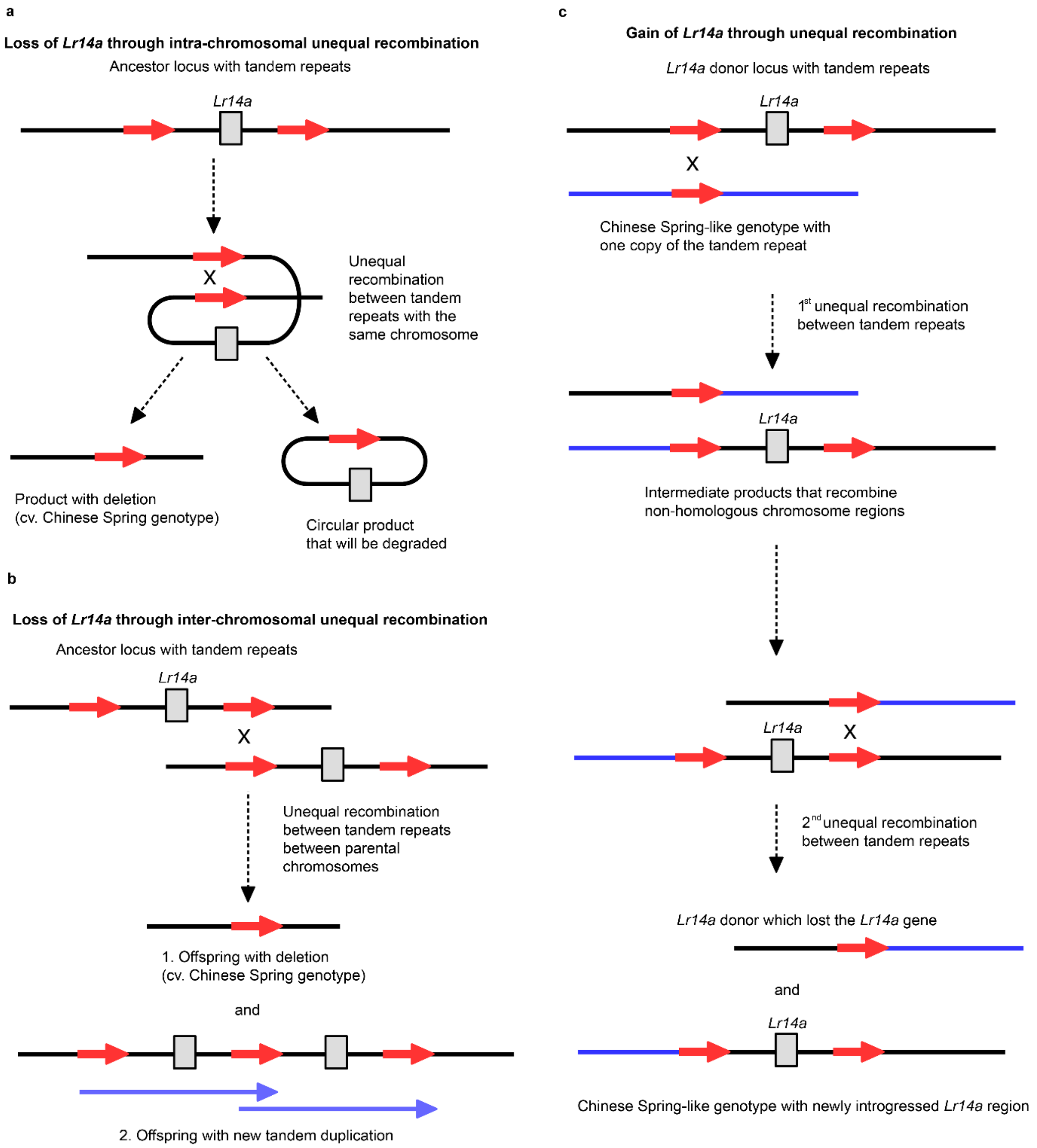

Supplementary Fig. 5. Evolutionary models for how tandem repeats (red arrows) can lead to the removal and gain of sequences. a, The tandem repeats of a single chromatid can align and serve as template for a recombination event. In this case, the reciprocal translocation results in a product where the sequence between the tandem repeats is deleted and a circular product that is subsequently lost. **b,** Tandem repeats align incorrectly during sexual recombination, leading to offspring carrying a deletion and offspring where the entire region between the tandem repeats is duplicated (blue arrows). **c,** Hypothetical model for how a foreign sequence can be introgressed. This requires the presence of tandem repeats flanking the foreign segment plus at least one copy of a homologous sequence at the acceptor site. This process requires two independent unequal recombination events and is thus less likely than those in **a** and **b**.

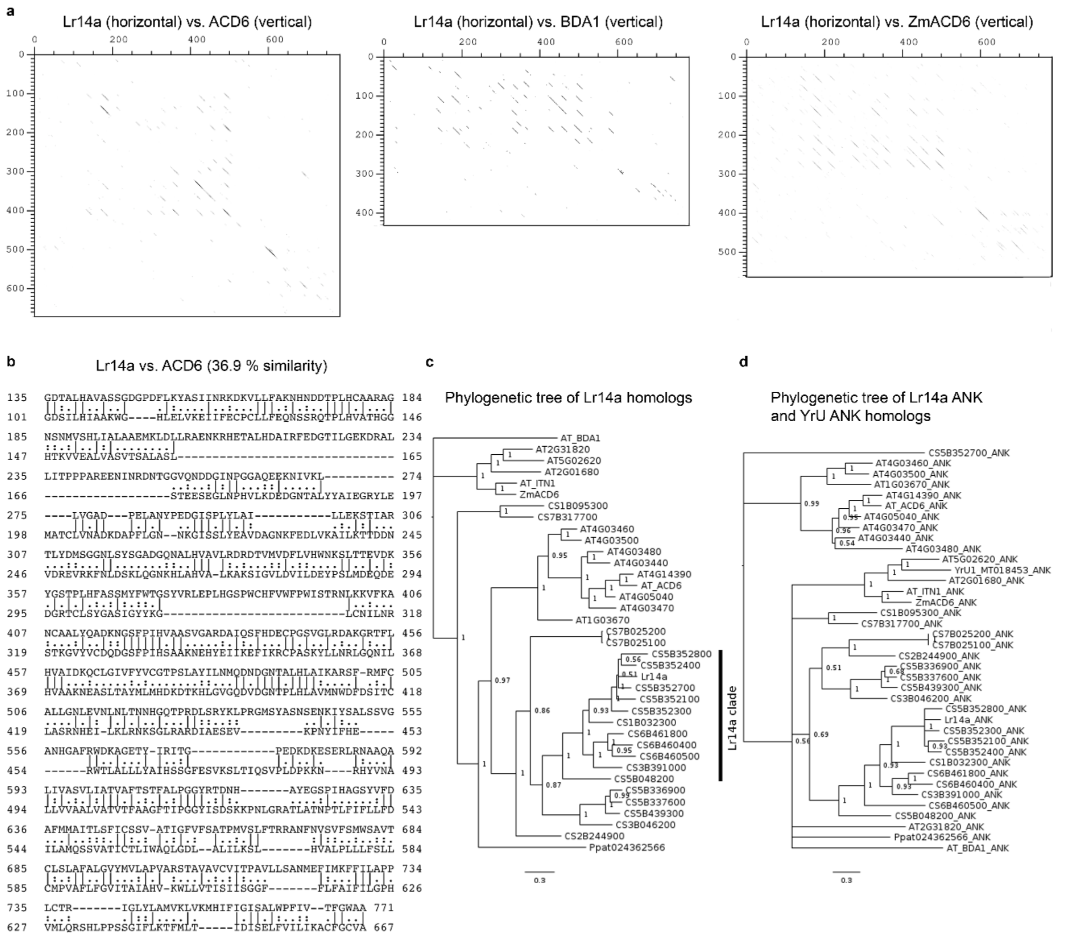

Supplementary Fig. 6. Comparative and phylogenetic analysis of LR14A homologs. a, Dotplot alignments of the LR14A protein with ACD6 and BDA1 from Arabidopsis and ZmACD6 from maize. Overall sequence organization is the same in all four proteins, despite a low level of sequence conservation. The repetitive structure of the ankyrin repeat domain in the N-terminal region of the protein (approximately positions 130-550) is reflected in the multiple short parallel stretches of sequence homology. **b,** Sequence alignment of LR14A protein with ACD6 from Arabidopsis shows 36.9% similarity. **c,** Phylogenetic tree of LR14A homologs from wheat Chinese Spring (prefix CS), Arabidopsis (prefix AT), and maize (prefix Zm). To construct the tree, three types of LR14A homologs were selected as follows: 1. the top ten hits of a blastp search of LR14A against Arabidopsis proteins, 2. the top ten hits of a blastp search of LR14A against all proteins encoded by the wheat (cv. Chinese Spring) B genome, and 3. the top ten hits of a blastp search of Arabidopsis ACD6 against all proteins encoded by the wheat (cv. Chinese Spring) B genome. The last search was performed to identify the closest homologs of ACD6 in wheat. The wheat dataset was reduced to the B genome to simplify the tree and shorten computation time. A and D genome homologs do not provide much additional information as they mostly cluster closely together with their B-genome orthologs. Note that LR14A is member of a distinct clade that has no Arabidopsis homologs. **d**, Phylogenetic tree of ANK repeat domain protein sequences of LR14A homologs from wheat Chinese Spring (prefix CS), Arabidopsis (prefix AT), and maize (prefix Zm), plus the recently published sequence of the yellow rust resistance protein YrU1[13]. The wheat, Arabidopsis and maize homologs are the same as those used for the phylogenetic tree in Supplementary Fig. 5c. Note that only the N-terminal part that contains the ANK repeat domain was used to construct the tree. The reason for that being that YrU1 does not have a transmembrane domain. Instead the ANK repeat domain is integrated in an NBS-LRR protein possibly as a decoy[13]. The phylogenetic tree shows that the ANK domain of YrU1 is clearly derived from an ANK-TM protein, suggesting that multiple ANK-TM proteins are involved in pathogenesis response.

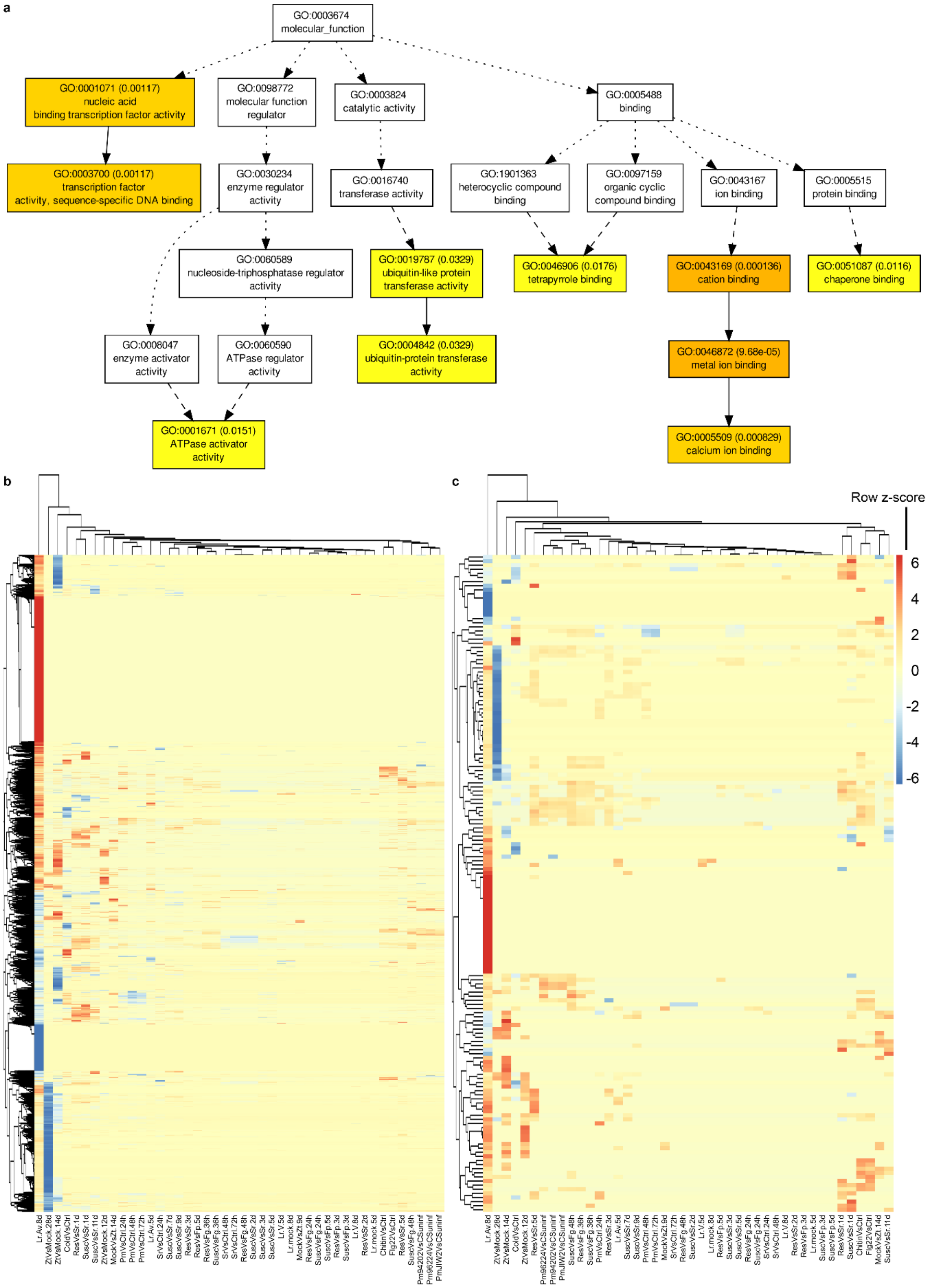
a
GO:0003674 molecular_function
GO:0001071 (0.00117) nucleic acid binding transcription factor activity
GO:0098772 molecular function regulator
GO:0003824 catalytic activity
GO:0005488 binding
GO:0003700 (0.00117) transcription factor activity, sequence-specific DNA binding
GO:0030234 enzyme regulator activity
GO:0016740 transferase activity
GO:1901363 heterocyclic compound binding
GO:0097159 organic cyclic compound binding
GO:0043167 ion binding
GO:0005515 protein binding
GO:0060589 nucleoside-triphosphatase regulator activity
GO:0019787 (0.0329) ubiquitin-like protein transferase activity
GO:0046906 (0.0176) tetrapyrrole binding
GO:0043169 (0.000136) cation binding
GO:0051087 (0.0116) chaperone binding
GO:0008047 enzyme activator activity
GO:0060590 ATPase regulator activity
GO:0004842 (0.0329) ubiquitin-protein transferase activity
GO:0046872 (9.68e-05) metal ion binding
GO:0001671 (0.0151) ATPase activator activity
GO:0005509 (0.000829) calcium ion binding
b
c
Row z-score
6
4
2
0
-2
-4
-6

Supplementary Fig. 7. Gene expression studies in *Nicotiana benthamiana* and comparisons of differentially expressed genes between different wheat treatments. a, Gene ontology (GO) enrichment analysis of *N. benthamiana* genes that were up-regulated in response to transient expression of *Lr14a* at 27 hours post infiltration. In the directed acyclic graph (DAG) the enriched GO pathways are depicted in yellow and orange. **b,** Heatmap of all differentially expressed genes (DEGs) between Thatcher*Lr14a* and Thatcher wheat cultivars, upon infection of an avirulent *P. triticina* isolate at 8 dpi. No other treatment shows a comparable induction or suppression of gene expression of the 7,986 DEGs. Log_2FC values were scaled to Z-scores, to improve data visualization. **c,** Heatmap of all DEGs, associated with "calcium ion binding" (GO:0005509), that were found between Thatcher*Lr14a* and Thatcher wheat cultivars, upon infection of an avirulent *P. triticina* isolate at 8 dpi. No other treatment shows a comparable induction or suppression of gene expression of the 160 DEGs. Log2FC values were scaled to Z-scores, to improve data visualization. The transcriptomic data for **b** and **c** were downloaded from wheat-expression.com[14-16] and the following stress treatments were considered: *Zymoseptoria tritici* (Zt; ERP009837), *Fusarium graminearum* (Fg; ERP013829), stripe rust and wheat powdery mildew (Sr, Pm; ERP013983, SRP041017), *Fusarium pseudograminearum* (Fp; SRP048912), PAMPs (chitin and flg22) and cold (SRP043554).

Supplementary Table 1. Leaf rust pathogen isolates and their infection types on different accessions.

Accession	Arina	Forno	Arina*LrFor*	Arina *Lr14a*	Thatcher *Lr14a*	Thatcher	Selkirk	Osmut	WW198 (Hevidi)	WW200 (Menceki)
Leaf rust isolate										
91047	3	3	3	3	3	3	3	2 ;	2	3
93012	3	2 ;	1 ;	1 2 ;	3 ;	3	1 ;	1 ;	3 ;	3
94015	3	3 ;	1 ;	1 ;	2 ;	3	1 ;	1 ;	2 ;	2
95001	3	2 ;	1 ;	2 ;	2 3 ;	3	1 ;	1 ;	3 ;	3
95012	3	3 ;	2 ;	3 ;	3 ;	3	2 ;	2 ;	3 ;	3
95037	3	3	3	3	3	3	1 ;	1 ;	1 ;	2 ;
96002	3	3 ;	1 ;	2 ;	2 ;	3	1 ;	2 ;	2 ;	3 ;
96007	3	3 ;	2 ;	2 ;	3 ;	3	3 ;	2 ;	3 ;	3 ;
96209	3	3 ;	1 ;	1 ;	3 ;	3	1 ;	3	1 ;	3 ;
96237	3	3 ;	1 ;	2 ;	2 ;	3	2 ;	2 ;	3	3
98014	3	2 ;	2 ;	2 ;	3 ;	3	1 ;	2 ;	3 ;	3

Note: Leaf rust (*Puccinia triticina*) isolates[1], available in our lab were used for seedling stage infection experiments on different accessions to characterize the virulence/avirulence formula of the *P. triticina* isolates against these accessions, containing (orange) or not containing (white) *Lr14a*. Thatcher*Lr14a* showed the same virulence/avirulence formula as Arina*LrFor* and Arina*Lr14a.* Also, Forno, the *Lr14a* donor line of Arina*LrFor* and Arina*Lr14a*, showed the same pattern while Arina and Thatcher, the susceptible parents of Arina*LrFor/14a* and Thatcher*Lr14a*, respectively, were susceptible. Selkirk, the donor Line of *Lr14a* in Thatcher*Lr14a* showed minor changes to the Thatcher*Lr14a* virulence/avirulence formula as well as the two durum (Hevidi and Menceki) and the spelt (Osmut) lines which showed a variation of virulence/avirulence patterns indicating the presence of different interaction factors of *Lr14a*[8-10,12]. Plants were infected ten days after sowing. Another ten days post inoculation phenotypes at seedling stage were defined.1 = small uredia with necrosis, 2 = small to medium sized uredia with necrosis or chlorosis, 3 = medium sized uredia with or without chlorosis, ; = hypersensitive flecks. Orange = *Lr14a* containing lines, blue = avirulent phenotype on *Lr14a*, red = virulent phenotype on *Lr14a*.

Supplementary Table 2. *Lr14a* haplotype segments.

Cultivar/accession	Chr.	Position	Ori[a]	comment
Arina*LrFor*	7B	9525576-9589766	for	Conserved in ArinaLrFor, Lancer and Spelt
Arina*LrFor*	7B	8800000-10206001	for	Conserved in ArinaLrFor and Chinese Spring
Chinese Spring	7B	748505600-749511601	for	Conserved in ArinaLrFor and Chinese Spring
Lancer	Un	93278164-93342513	for	Conserved in ArinaLrFor, Lancer and Spelt
Spelt	7B	722604200-722542053	rev	Conserved in ArinaLrFor, Lancer and Spelt

[a] Orientation relative to *Lr14a* locus in Arina*LrFor* (for = forward, rev = reverse).

Chromosomal locations of haplotype segments with similarity to the *Lr14a* locus that were used for comparative analyses and molecular dating.

Supplementary Table 3. Top 10 hits of HHPred analysis of the LR14A protein.

PDB entry[a]	Description	E-value	Ref[b]
5Y4D_A	ANK2, Homo sapiens	9.60E-54	1[17]
6PQQ_D	Transient receptor potential cation channel TRPA1, Homo sapiens	4.20E-52	2[18]
5LEB_A	DARPin DDD_D12_06_D12_06_D12, biosynthetic protein	1.50E-51	3[19]
5Y4D_A	ANK2, Homo sapiens	3.90E-51	1[17]
1N11_A	D34 region of Ankyrin-R, Homo sapiens	8.10E-51	4[20]
3J9P_A	Transient receptor potential cation channel TRPA1t, Homo sapiens	6.60E-49	5[21]
4CJ9_B	BurrH DNA-binding protein, Burkholderia rhizoxinica	5.30E-51	6[22]
4RLV_A	ANK repeat AnkB, Homo sapiens	1.10E-48	7[23]
6MOL_A	Monoextended DARPin M_R12, biosynthetic protein	9.50E-48	8[24]
4CJ9_B	BurrH DNA-binding protein, Burkholderia rhizoxinica	2.20E-50	6[22]

[a] Protein Data Bank (rcsb.org) entry.

[b] Reference to original publication on the respective 3D structure.

Collection of descriptions and related references of the top ten HHPred analysis hits of the LR14A protein.

Supplementary Table 4. *Lr14a* molecular dating.

**Sequece1**	**Sequence2**	**Aligned**[a]	**Idenity**[b]	**TI**[c]	**TV**[d]	**Divg.**[e]	**STD**[f]
Arina*LrFor*	LancerUn	48662	99.88%	29	27	0.044	0.01
Arina*LrFor*	Spelt	48658	99.97%	9	4	0.01	0
Arina*LrFor*	Chinese Spring	247056	99.95%	81	37	0.018	0

[a] Number of bp aligned for the divergence time estimate.

[b] Sequence identity.

[c] Number of transitions.

[d] Nmber of transversions.

[e] Estimated divergence in million years.

[f] Standard deviation of estimate.

Molecular dating of divergence times of *Lr14a*-like wheat haplotypes. Divergence time estimates are based on alignments of intergenic sequences (e.g. regions that are presumably free from selection pressure).

Supplementary Table 5. PCR confirmation of *Lr14a* region haplotype breakpoints.

5' breakpoint		expected bp	amplified bp[a]	3' breakpoint		expected bp	amplified bp[a]
MCK268_break5_AC	MCK271_break5_A	306	0	MCK277_break3_AC	MCK280_break3_A	529	600
	MCK272_break5_A	418	500		MCK281_break3_A	496	550
	MCK273_break5_A	514	600		MCK282_break3_A	392	400
MCK269_break5_AC	MCK271_break5_A	257	0	MCK278_break3_AC	MCK280_break3_A	581	600
	MCK272_break5_A	369	450		MCK281_break3_A	548	550
	MCK273_break5_A	465	550		MCK282_break3_A	444	500
MCK270_break5_AC	MCK271_break5_A	189	0	MCK279_break3_AC	MCK280_break3_A	682	650
	MCK272_break5_A	310	400		MCK281_break3_A	649	600
	MCK273_break5_A	397	0		MCK282_break3_A	545	550

[a] agarose gel estimation base pairs (bp)

green = expected length validated by sequencing

Amplification of Arina*LrFor* breakpoints (one primer inside, one primer outside of *Lr14a* segment missing in Chinese Spring) with different primer combinations. PCR amplicons run at the expected sizes on an agarose gel, sequencing of representative amplicons (green) validated length and therefore the correct positioning of the *Lr14a* region in Arina*LrFor*.

Supplementary Note 1. The *Lr14a* leaf rust resistance gene in hexaploid bread wheat and tetraploid durum wheat

Genetic identification of *Lr14* alleles

The *Lr14a* gene was introduced originally to the bread wheat gene pool as an introgression from the tetraploid Yaroslav emmer wheat by McFadden in the year 1930[29]. The first bread wheat cultivar (cv.) with *Lr14a* was cv. "Hope" that was subsequently used for crosses in many breeding programs mostly because of the presence of the *Sr2* locus in this cultivar, providing resistance to stem rust. The *Lr14a* gene was first described by McIntosh et al. 1967[30] as an incompletely dominant gene, although the same gene showed dominant inheritance in a different genetic source, which was attributed to the genetic background[9]. *Lr14a* confers a mesothetic phenotype (X type of reaction) which is characterized by the presence of two or more different infection types (IT) on a single leaf[8]. The gene was mapped on wheat chromosome 7B and was linked with genes for mildew and stem rust resistance[3]. A leaf rust resistance gene in the variety Maria Escobar also showed an X phenotype to different *Puccinia triticina* isolates and was found to be closely linked to *Lr14a*. It was described as allelic to *Lr14a* although recombination was observed between *Lr14a* and *Lr14b*[9], resulting in some uncertainty on the interpretation of the genetic data. In the same study, the authors analyzed the inheritance of virulence in the leaf rust pathogen to both *Lr14a* and *Lr14b* and found different loci for avirulence in the pathogen. Using the gene-specific marker for *Lr14a* developed in this study, we did not get an amplification on Thatcher*Lr14b* DNA (Supplementary Fig. 3a). Furthermore, no amplification of the gene was found using the primers for gene amplification (Supplementary Data 9). Phenotypically, Thatcher*Lr14b* was susceptible when infected with the *Lr14a* avirulent *P. triticina* isolate 96209 (Supplementary Fig. 3c).

In durum wheat, the *Lr14a* gene was described as a race-specific resistance gene conferring near immunity under field conditions[31]. The *Lr14a* gene in durum wheat was acting as a recessive or dominant gene and the expression depended on test conditions and genetic background. The source of this gene was the Chilean durum wheat variety Llareta. Marone et al.[11] identified a leaf rust resistance gene in cultivar Creso at the same map position. The two donor varieties Llareta and Creso have different pedigrees, but they both contain a gene at the map position of *Lr14a*. Therefore, the gene in Creso was considered an allele of *Lr14a* and called *Lr14c*. In a later study[32], it was questioned if it is really an allele of *Lr14a* or *Lr14a* together with a gene conveying a minor modifying effect on the resistance in Creso. Indeed, Creso showed a strong resistance comparable to the one observed in Arina*LrFor* (Supplementary Fig. 3c). The *Lr14c* gene from Creso was identified by the *Lr14a* gene-specific primer (Supplementary Data 9), and sequencing revealed a coding sequence with 100% identity between the Creso and the Arina*LrFor* *Lr14a* gene. Another potential allele of *Lr14a* was described in the Chinese wheat cv. Bimai 16 and called *LrBi16*[33].

Genetic mapping of *Lr14a*

Based on molecular markers, Herrera-Foessel et al.[34] mapped the *Lr14a* gene on chromosome 7B close to the microsatellite markers *Xgwm344* and *Xgwm146*. Depending on the mapping population studied, these two markers either flanked the *Lr14a* gene, or they were both located proximally to the gene[11,35]. Despite the slightly different map positions, in all these studies the two markers consistently mapped close to *Lr14a* or *Lr14c*. Based on this and other genetic mapping studies on *Lr14a*, Terraciano et al.[7] developed additional markers for the gene that are useful for resistance breeding.

Lr14a expression is dependent on environmental conditions and the genetic background

Law and Johnson[10] performed a detailed genetic analysis of *Lr14a* resistance expression in bread wheat mostly based on chromosome substitution lines in the bread wheat genotype Chinese Spring. Using crossing experiments, at least four loci modifying *Lr14a* action were identified: one modifier was located on the short arm of 7B (the same chromosome where *Lr14a* is located on the long arm). Furthermore, the homoeologous chromosomes 7A and 7D were both identified to carry modifying loci. Chromosome 7A in cv. Hope carries a gene or genes that increases resistance, whereas chromosome 7D of cv. Hope has one or several genes that lower resistance. It was speculated that these modifiers are homoeologous genes to the modifier on chromosome 7BS of cv. Hope, but this has not been clarified. The modifier loci did not show a detectable phenotype in the absence of *Lr14a*. However, particular combinations of the modifier loci resulted in completely susceptible *Lr14a* lines, thus suppressing *Lr14a* gene activity. Such susceptible genotypes were homozygous for the Hope 7B chromosome[10]. Thus, in this study at least four loci were identified to enhance or suppress *Lr14a* resistance.

Genotype-specific resistance phenotypes for *Lr14a* were also described by Dyck and Samborski[9]. Whereas Thatcher*Lr14a* (a near isogenic line of Thatcher containing *Lr14a*) showed an X infection type for three different *P. triticina* isolates, cultivar Selkirk, also containing *Lr14a* showed infection types between 1 and 2 for the same three isolates.

In the same study[9] the expression of leaf rust resistance in cv. Hope, later defined as the *Lr14a* gene, was found to vary according to environment. This was also described by Law and Johnson[10] and seems to be based on temperature dependence of gene function as well as possible additional environmental factors.

Race-specific vs. quantitative nature of the *Lr14a* gene

As described in the early studies[8-10,30] *Lr14a* is a race-specific resistance gene with corresponding avirulence determinants in the leaf rust pathogen. However, the strong influence of genetic modifiers and the environmental dependence result in differential resistance expression and varying phenotypes, which is also a characteristic of quantitatively acting resistance genes. The quantitative nature of resistance conferred by *Lr14a* is also illustrated by the genotypes Arina*LrFor* vs. Arina*Lr14a* where the earlier has a stronger *Lr14a*-based phenotype possibly because of differential modifier genes in the two genetic backgrounds (Arina*LrFor* was backcrossed twice (BC2) while Arina*Lr14a* was backcrossed three times and, therefore, has less genetic background of the *Lr14a* donor Forno). Such additive gene interactions are characteristic for quantitatively acting rust resistance genes which are used to achieve durable resistance in breeding programs[36].

All these observations, as well as the unique mesothetic phenotype with different infection types on the same leaf, make *Lr14a* a highly interesting target for molecular identification of the gene and the study of its possible function.

Agricultural use of *Lr14a*

After its introduction into the bread wheat gene pool in 1930[1], *Lr14a* was used intensively in Canada after 1937. *P. triticina* isolates virulent on *Lr14a* started to dominate in Canada in the 1950s and the importance of *Lr14a* decreased[37-40]. *Lr14a* containing wheat cultivars were used in breeding programs around the globe. Different studies show the presence of *Lr14a* in wheat lines in Europe[41-43], India[44], China[45,46], and in the USA[47]. However, in India, China, Canada, at the US American east coast[48], and in Mexico[49], *Lr14a* was mostly overcome[8].

In durum wheat, *Lr14a* is present and effective against leaf rust and is broadly used. Up to 95% of CIMMYT durum wheat lines contain *Lr14a* and resistance strategies seem generally built on this gene in the Mediterranean area[35,50]. But first virulent *P. triticina* isolates on *Lr14a* were also reported from durum in France[32], Spain[51], and Morocco[52]. However, avirulent *P.*

triticina isolates were described in the great plains of the USA[53], suggesting that *Lr14a* might still be useful in modern resistance breeding in certain regions, particularly in combination with other *Lr* resistance genes, and modifying genes, as indicated also by durable leaf rust resistance of cultivar Forno[6].

Supplementary Note 2. *Lr14a* in spelt lines

The *Lr14a*-containing spelt lines (Supplementary Fig. 3d) showed no characteristic mesothetic reactions with the Arina*LrFor* avirulent *P. triticina* isolate 96209. However, with different other *P. triticina* isolates, among them *P. triticina* isolates that are virulent on Arina*LrFor*, the characteristic mesothetic reaction was observed (Supplementary Table 1). This suggests either the presence of an additional leaf rust resistance gene with different race specificity or, more likely, the presence of unknown modifiers not only influencing the quantitative phenotype but also the specificity of the reaction to different virulent and avirulent *P. triticina* isolates. Crosses between three spelt lines and the susceptible bread wheat cultivar Arina produced F_1 plants with stronger seedling resistance (including mesothetic responses) after Infection with *P. triticina* isolate 96209 than either parents (Supplementary Fig. 3e), providing evidence for the presence of *Lr14a* modifiers[8-10].

Supplementary Note 3. Haplotype diversity at the *Lr14a* locus in wheat

We analyzed chromosome-scale assemblies of ten wheat cultivars and one spelt line that were produced in the framework of the recently published 10+ wheat genome project[54] for presence of *Lr14a*. In addition to Arina*LrFor*, we found the *Lr14a* gene only in wheat cultivar Lancer and in spelt PI190962. The *Lr14a* gene sequence was identical in all three genotypes. Although the Lancer and spelt assemblies are fragmented in the *Lr14a* region, a 64.1 kb sequence containing *Lr14a* could be aligned in all three genomes (Supplementary Table 2). Molecular dating indicates that the Lancer and Arina*LrFor* haplotypes diverged approximately 44,000 years ago (Supplementary Table 4). Interestingly, spelt has a more similar haplotype which diverged from the one in Arina*LrFor* approximately 8,000-12,000 years ago. Chinese Spring was the only wheat cultivar where we found extensive sequences that could be aligned over several hundred kb with Arina*LrFor* at the *Lr14a* locus (Fig. 3a). However, a ~315 kb region containing *Lr14a* and at least two more genes are completely absent from Chinese Spring. The breakpoints of this insertion/deletion in Arina*LrFor* were confirmed by PCR and sequencing (Supplementary Table 5). The ~315 kb region is flanked by a series of large (10-50 kb) tandem repeats. This sequence organization suggests that the ancestral locus containing the *Lr14a* segment was deleted in the Chinese Spring haplotype through unequal crossing over between the tandem repeats (Fig. 3b, Supplementary Fig. 5). Molecular dating indicates that the Chinese Spring and *Lr14a* haplotypes diverged approximately 18,000 years ago (Supplementary Table 4). Thus, in the lineage leading to Chinese Spring, the *Lr14a* containing segment was deleted but the haplotype segment overall retained.

The fact that no sequences could be aligned with the *Lr14a* region of Arina*LrFor* in the other sequenced recently published wheat cultivars[54] indicates a strong haplotype diversity at this locus where sequence collinearity completely breaks down between the cultivars. Furthermore, the fact that two of the sequenced wheat genomes contain haplotype segments which are similar to, but not identical with the *Lr14a* locus in Arina*LrFor* suggests that multiple *Lr14a*-like haplotypes were introgressed independently into the bread wheat germplasm.

Supplementary References

1. Messmer, M.M. *et al.* Genetic analysis of durable leaf rust resistance in winter wheat. *Theoretical and Applied Genetics* 100, 419-431 (2000).
2. Peterson, R.F., Campbell, A.B. & Hannah, A.E. A Diagrammatic scale for estimating rust intensity on leaves and stems of cereals. *Canadian Journal of Research Section C-Botanical Sciences* 26, 496-& (1948).
3. Mosavi, L.K., Minor, D.L., Jr. & Peng, Z.Y. Consensus-derived structural determinants of the ankyrin repeat motif. *Proc Natl Acad Sci U S A* 99, 16029-34 (2002).
4. Dong, X. The role of membrane-bound ankyrin-repeat protein ACD6 in programmed cell death and plant defense. *Sci STKE* 2004, pe6 (2004).
5. Lu, H., Liu, Y. & Greenberg, J.T. Structure-function analysis of the plasma membrane-localized Arabidopsis defense component ACD6. *Plant Journal* 44, 798-809 (2005).
6. Schnurbusch, T. *et al.* Dissection of quantitative and durable leaf rust resistance in Swiss winter wheat reveals a major resistance QTL in the Lr34 chromosomal region. *Theor Appl Genet* 108, 477-84 (2004).
7. Terracciano, I. *et al.* Development of COS-SNP and HRM markers for high-throughput and reliable haplotype-based detection of Lr14a in durum wheat (Triticum durum Desf.). *Theor Appl Genet* 126, 1077-101 (2013).
8. McIntosh, R.A., Wellings, C.R. & Park, R.F. *Wheat rusts: an atlas of resistance genes*, (Springer Netherlands, 1995).
9. Dyck, P.L. & Samborski, D.J. The genetics of two alleles for leaf rust resistance at the *Lr14* locus in wheat. *Canadian Journal of Genetics and Cytology* 12, 689-694 (1970).
10. Law, C.N. & Johnson, R. A genetic study of leaf rust resistance in wheat. *Canadian Journal of Genetics and Cytology* 9, 805-822 (1967).
11. Marone, D. *et al.* Genetic analysis of durable resistance against leaf rust in durum wheat. *Molecular Breeding* 24, 25-39 (2009).
12. Dyck, P. & Johnson, R. Temperature sensitivity of genes for resistance in wheat to Puccinia recondita. *Canadian Journal of Plant Pathology* 5, 229-234 (1983).
13. Wang, H., Zou, S., Li, Y., Lin, F. & Tang, D. An ankyrin-repeat and WRKY-domain-containing immune receptor confers stripe rust resistance in wheat. *Nature Communications* 11, 1353 (2020).
14. Ramirez-Gonzalez, R.H. *et al.* The transcriptional landscape of polyploid wheat. *Science* 361, eaar6089 (2018).
15. Ma, X., Keller, B., McDonald, B.A., Palma-Guerrero, J. & Wicker, T. Comparative transcriptomics reveals how wheat responds to infection by *Zymoseptoria tritici*. *Mol Plant Microbe Interact* 31, 420-431 (2018).
16. Praz, C.R. *et al.* Non-parent of origin expression of numerous effector genes indicates a role of gene regulation in host adaption of the hybrid triticale powdery mildew pathogen. *Front Plant Sci* 9, 49 (2018).
17. Chen, K., Li, J., Wang, C., Wei, Z. & Zhang, M. Autoinhibition of ankyrin-B/G membrane target bindings by intrinsically disordered segments from the tail regions. *Elife* 6(2017).
18. Suo, Y. *et al.* Structural insights into electrophile iIrritant sensing by the human TRPA1 channel. *Neuron* 105, 882-894 e5 (2020).
19. Wu, Y. *et al.* Rigidly connected multispecific artificial binders with adjustable geometries. *Sci Rep* 7, 11217 (2017).

20. Michaely, P., Tomchick, D.R., Machius, M. & Anderson, R.G. Crystal structure of a 12 ANK repeat stack from human ankyrinR. *Embo j* 21, 6387-96 (2002).
21. Paulsen, C.E., Armache, J.P., Gao, Y., Cheng, Y. & Julius, D. Structure of the TRPA1 ion channel suggests regulatory mechanisms. *Nature* 520, 511-7 (2015).
22. Stella, S. *et al.* BuD, a helix-loop-helix DNA-binding domain for genome modification. *Acta Crystallogr D Biol Crystallogr* 70, 2042-52 (2014).
23. Wang, C. *et al.* Structural basis of diverse membrane target recognitions by ankyrins. *Elife* 3(2014).
24. Mohan, K. *et al.* Topological control of cytokine receptor signaling induces differential effects in hematopoiesis. *Science* 364(2019).
25. Pont, C. *et al.* Tracing the ancestry of modern bread wheats. *Nature Genetics* 51, 905-911 (2019).
26. Loutre, C. *et al.* Two different CC-NBS-LRR genes are required for Lr10-mediated leaf rust resistance in tetraploid and hexaploid wheat. *The Plant Journal* 60, 1043-1054 (2009).
27. Yahiaoui, N., Kaur, N. & Keller, B. Independent evolution of functional *Pm3* resistance genes in wild tetraploid wheat and domesticated bread wheat. *The Plant Journal* 57, 846-856 (2009).
28. Zhang, W. *et al.* Identification and characterization of Sr13, a tetraploid wheat gene that confers resistance to the Ug99 stem rust race group. *Proceedings of the National Academy of Sciences* 114, E9483-E9492 (2017).
29. McFadden, E.S. A successful transfer of emmer characters to Vulgare wheat. *Agronomy journal* 12, pp. 1020-1034 (1930).
30. Mcintosh, R.A., Luig, N.H. & Baker, E.P. Genetic and cytogenetic studies of stem rust leaf rust and powdery mildew resistances in Hope and related wheat cultivars. *Australian Journal of Biological Sciences* 20, 1181-1192 (1967).
31. Herrera-Foessel, S.A., Singh, R.P., Huerta-Espino, J., Yuen, J. & Djurle, A. New genes for leaf rust resistance in CIMMYT durum wheats. *Plant Disease* 89, 809-814 (2005).
32. Goyeau, H., Ammar, K. & Berder, J. Virulence in *Puccinia triticina* for durum wheat cultivar Creso and other durum wheat cultivars carrying resistance gene *Lr14a* in France. in *Plant Dis* 2010/08/01 edn Vol. 94 1068 (2010).
33. Zhang, P.P., Zhou, H.X., Lan, C.X., Li, Z.F. & Liu, D.Q. An AFLP marker linked to the leaf rust resistance gene LrBi16 and test of allelism with Lr14a on chromosome arm 7BL. *Crop Journal* 3, 152-156 (2015).
34. Herrera-Foessel, S.A. *et al.* Identification and molecular characterization of leaf rust resistance gene *Lr14a* in durum wheat. *Plant Dis* 92, 469-473 (2008).
35. Maccaferri, M. *et al.* A major QTL for durable leaf rust resistance widely exploited in durum wheat breeding programs maps on the distal region of chromosome arm 7BL. *Theor Appl Genet* 117, 1225-40 (2008).
36. Singh, R.P. *et al.* Disease impact on wheat yield potential and prospects of genetic control. *Annual Review of Phytopathology* 54, 303-22 (2016).
37. Kolmer, J.A. Evolution of distinct populations of *Puccinia pecondita* f. sp. tritici in Canada. *Phytopathology* 81, 316-322 (1991).
38. Liu, J.Q. & Kolmer, J.A. Genetics of leaf rust resistance in Canadian spring wheats AC Domain and AC Taber. *Plant Dis* 81, 757-760 (1997).
39. McCallum, B.D., Fetch, T. & Chong, J. Cereal rust control in Canada. *Australian Journal of Agricultural Research* 58, 639-647 (2007).

40. Martens, J.W. & Dyck, P.L. Genetics of resistance to rust in cereals from a Canadian perspective. *Canadian Journal of Plant Pathology* 11, 78-85 (1989).
41. Hysing, S.C. *et al.* Leaf rust (*Puccinia triticina*) resistance in wheat (*Triticum aestivum*) cultivars grown in Northern Europe 1992-2002. *Hereditas* 143, 1-14 (2006).
42. Pathan, A.K. & Park, R.F. Evaluation of seedling and adult plant resistance to leaf rust in European wheat cultivars. *Euphytica* 149, 327-342 (2006).
43. Bartoš, P. *et al.* Achievements and prospects of wheat breeding for disease resistance. *Czech Journal of Genetics and Plant Breeding* 38, 16-28 (2012).
44. Saini, R.G. *et al.* Genes Lr48 and Lr49 for hypersensitive adult plant leaf rust resistance in wheat (*Triticum aestivum* L.). *Euphytica* 124, 365-370 (2002).
45. Singh, R.P., Chen, W.Q. & He, Z.H. Leaf rust resistance of spring, facultative, and winter wheat cultivars from China. *Plant Dis* 83, 644-651 (1999).
46. Li, Z.F. *et al.* Seedling and slow rusting resistance to leaf rust in Chinese wheat cultivars. *Plant Dis* 94, 45-53 (2010).
47. Oelke, L.M. & Kolmer, J.A. Characterization of leaf rust resistance in Hard Red spring wheat cultivars. *Plant Dis* 88, 1127-1133 (2004).
48. Kolmer, J.A. Virulence Phenotypes of *Puccinia triticina* in the South Atlantic states in 1999. *Plant Dis* 86, 288-291 (2002).
49. Zhang, J.X. *et al.* Genetics of leaf rust resistance in brambling wheat. *Plant Dis* 92, 1111-1118 (2008).
50. Loladze, A., Kthiri, D., Pozniak, C. & Ammar, K. Genetic analysis of leaf rust resistance in six durum wheat genotypes. *Phytopathology* 104, 1322-8 (2014).
51. Soleiman, N.H. *et al.* Short communication: Emergence of a new race of leaf rust with combined virulence to *Lr14a* and *Lr72* genes on durum wheat. *Spanish Journal of Agricultural Research* 14, e10SC02 (2016).
52. Aoun, M. *et al.* Genotyping-by-sequencing for the study of genetic diversity in *Puccinia triticina. Plant Dis* 104, 752-760 (2020).
53. Ordonez, M.E. & Kolmer, J.A. Differentiation of molecular genotypes and virulence phenotypes of *Puccinia triticina* from common wheat in North America. *Phytopathology* 99, 750-8 (2009).
54. Walkowiak, S. *et al.* Multiple wheat genomes reveal global variation in modern breeding. *Nature* (2020).
