# Supplementary material for: A membrane-bound ankyrin repeat protein confers race-specific leaf rust disease resistance in wheat
Source: Nat Commun. 2021 Feb 11;12:956. doi: 10.1038/s41467-020-20777-x (PMC7878491; doi:10.1038/s41467-020-20777-x)
Supplement: Supplementary file 2 — Reporting Summary [file 41467_2020_20777_MOESM2_ESM.pdf]

nature research

Corresponding author(s): Markus C. Kolodziej, Thomas Wicker, Simon Krattinger, Beat Keller

Last updated by author(s): Dec 15, 2020

Reporting Summary

Nature Research wishes to improve the reproducibility of the work that we publish. This form provides structure for consistency and transparency in reporting. For further information on Nature Research policies, seeAuthors & Referees and theEditorial Policy Checklist .

Statistics

For all statistical analyses, confirm that the following items are present in the figure legend, table legend, main text, or Methods section.

n/a	Confirmed	
☐	☒	The exact sample size (*n*) for each experimental group/condition, given as a discrete number and unit of measurement
☐	☒	A statement on whether measurements were taken from distinct samples or whether the same sample was measured repeatedly
☐	☒	The statistical test(s) used AND whether they are one- or two-sided<br>*Only common tests should be described solely by name; describe more complex techniques in the Methods section.*
☒	☐	A description of all covariates tested
☒	☐	A description of any assumptions or corrections, such as tests of normality and adjustment for multiple comparisons
☐	☒	A full description of the statistical parameters including central tendency (e.g. means) or other basic estimates (e.g. regression coefficient) AND variation (e.g. standard deviation) or associated estimates of uncertainty (e.g. confidence intervals)
☐	☒	For null hypothesis testing, the test statistic (e.g. *F*, *t*, *r*) with confidence intervals, effect sizes, degrees of freedom and *P* value noted<br>*Give P values as exact values whenever suitable.*
☒	☐	For Bayesian analysis, information on the choice of priors and Markov chain Monte Carlo settings
☒	☐	For hierarchical and complex designs, identification of the appropriate level for tests and full reporting of outcomes
☒	☐	Estimates of effect sizes (e.g. Cohen's *d*, Pearson's *r*), indicating how they were calculated

Our web collection on statistics for biologists contains articles on many of the points above.

Software and code

Policy information about availability of computer code

Data collection	Bioinformatic data were generated/collected using Ubuntu v. 18 and MutChromSeq (https://github.com/steuernb/MutChromSeq), Mikroscopy: LAS AF Version 2.7.3.9723 Leica Microsystems CMS GmbH. Imaging: Fusion FX Imaging System (Vilber Lourmat, Eberhardzell, Germany), Epson Perfection V850 Pro scanner (Epson, Kloten, Switzerland)
Data analysis	CLC Main Workbench v. 20.0.2, R pagage GOseq v. 1.38.0, AgriGo v. 2.0, Fiji v. (variable e.g. 1.52t), CFX Manager Software 3.1, FastQC v. 0.11.7, cutadapt v. (from Martin, EMBnet.Journal 2011), sickle v. 1.33, perl v.5.26.1, dwgsim v. 0.1.11-3build1, Clustalx v. 2.1, Clustalw v. 2.1, water v. 6.6.0, HHPred v. 3.0 (Zimmermann et. al. J Mol Biol. 2018), dotter v. 4.44.1, MrBayes v. 3.2.6, FigTree v. 1.4.4, blast (blastall v. 0.66-3), RaptorX v. 4.0, Phyre2 v. 2.0, TMHMM server v. 2.0, Pileup2XML and MutChromSeq v. (both from Sánchez-Martín et. al. Genome Biol 2016), RStudio v. 1.2.5019

For manuscripts utilizing custom algorithms or software that are central to the research but not yet described in published literature, software must be made available to editors/reviewers. We strongly encourage code deposition in a community repository (e.g. GitHub). See the Nature Research guidelines for submitting code & software for further information.

Data

Policy information about availability of data

All manuscripts must include a data availability statement. This statement should provide the following information, where applicable:

- Accession codes, unique identifiers, or web links for publicly available datasets
- A list of figures that have associated raw data
- A description of any restrictions on data availability

All data supporting the findings of this work are available within the paper and its supplementary information files. Sequence data were deposited at the NCBI GenBank under the accession number MT123593 (https://www.ncbi.nlm.nih.gov/nuccore/MT123593.1/) (Lr14a coding sequence), and at the NCBI sequence read archive (SRA) database under the accession number PRJNA529355 (https://www.ncbi.nlm.nih.gov/sra/?term=PRJNA529355) (flow-sorted chromosome 7B of three ArinaLrFor Lr14a EMS mutants) or are available via the 10+ Wheat Genome Project9 (www.10wheatgenomes.com, https://wheat.ipk-gatersleben.de//). RNAseq raw data were deposited at the NCBI SRA database under the accession number PRJNA674985 (https://www.ncbi.nlm.nih.gov/sra/?term=PRJNA674985) for Triticum

aestivum or PRJNA674843 (https://www.ncbi.nlm.nih.gov/sra/?term=PRJNA674843) for Nicotiana benthamiana. A reporting summary for this article is available as a supplementary information file. The datasets and plant materials generated and analyzed during the current study are available from the corresponding author upon request. The source data underlying figures 1a,b,d,e, 2, 4 and 5c,d, as well as supplementary figures 1, 3 and 4a,b,c,d are provided as a Source Data file.

Field-specific reporting

Please select the one below that is the best fit for your research. If you are not sure, read the appropriate sections before making your selection.

☒ Life sciences ☐ Behavioural & social sciences ☐ Ecological, evolutionary & environmental sciences

For a reference copy of the document with all sections, see nature.com/documents/nr-reporting-summary-flat.pdf

Life sciences study design

All studies must disclose on these points even when the disclosure is negative.

Sample size	For designed experiments, at least 3 biological replicates were generated to ensure statistical interpretability. For experiments with more than 3 samples, space, costs and handling were the major concerns in defining the number of samples. Sample numbers are always part of figure/ table legend.
Data exclusions	outlier of qPCR measurements due to wrong or no amplification were excluded from the analysis. One outlier from the SA measurements was excluded because it was obvious that something with the sample preparation/measurement went wrong because value was several times higher than other 4 replicates.
Replication	If possible, experiments were repeated 3+ times (e.g. Infection at field and seedling stage, VIGS, Nicotiana transformations). Repetitions varied in intensity/strength (e.g. field infections due to environmental variations) but always resulted in the same major result. Replications which failed completely, because of e.g. missing infection, were not analyzed. If repetition was not possible and also if it was possible, 3+ biological replicates were taken/measured/generated in same experiments (e.g. expression, SA measurements)
Randomization	Single plots in field replicates were ordered in a randomized way, phenotypes were taken "blind" also for seedling phenotypes. Leaf samples (e.g. for expression, SA measurement) were taken from random plants on same tray. Random Nicotiana bentamiana plants were transient transformed on rotating positions (different constructs) on same developed leaves. For height measurements, random 10 plants / plot in 3 replicates were taken. for plant weight, all plants grown in rotating rows were sampled.
Blinding	phenotyping of disease responses were performed blinded. Samples for other experiments were sampled randomized, but not blinded to ensure sampling of the right samples. Analysis of experiment outcomes (sampled samples, imaging of Nicotiana leaves and analyzing phenotypes) were sorted by numbers and later after analysis compared with the sample identity.

Reporting for specific materials, systems and methods

We require information from authors about some types of materials, experimental systems and methods used in many studies. Here, indicate whether each material, system or method listed is relevant to your study. If you are not sure if a list item applies to your research, read the appropriate section before selecting a response.

Materials & experimental systems

n/a	Involved in the study	
☒	☐	Antibodies
☒	☐	Eukaryotic cell lines
☒	☐	Palaeontology
☒	☐	Animals and other organisms
☒	☐	Human research participants
☒	☐	Clinical data

Methods

n/a	Involved in the study	
☒	☐	ChIP-seq
☒	☐	Flow cytometry
☒	☐	MRI-based neuroimaging
