# Supplementary material for: A membrane-bound ankyrin repeat protein confers race-specific leaf rust disease resistance in wheat
Source: Nat Commun. 2021 Feb 11;12:956. doi: 10.1038/s41467-020-20777-x (PMC7878491; doi:10.1038/s41467-020-20777-x)
Supplement: Supplementary file 3 — Description of Additional Supplementary Files [file 41467_2020_20777_MOESM3_ESM.pdf]

**Description of Additional Supplementary Files**

**Supplementary Data 1** Genotyping and phenotyping results of Arina X Forno F5 RIL population. Genotype and phenotype of *Lr14a* containing/not containing RILs co-segregated. Infection was performed ten days after sowing, phenotyping was done ten days post inoculation with avirulent *Puccinia triticina* isolate 96209. Red = 0/S = *Lr14a* marker null allele/susceptible = no hypersensitive flecks; Blue = 1/R = *Lr14a* marker detection of *Lr14a* presence/resistant = presence of hypersensitive flecks.

**Supplementary Data 2** Plant material used in this study and *Lr14a* genotyping results. Table of plants, available in our lab, used for genotypic and partially phenotypic analysis of *Lr14a* presence and allele mining. *Lr14a* marker 0 = *Lr14a* not present (red), 1 = *Lr14a* present (blue). Reference numbers in table: 1[25], 2[26], 3[27].

**Supplementary Data 3** Results of the RNAseq analysis in Thatcher/Thatcher*Lr14a* wheat after transient expression of *Lr14a*. Gene ontology (GO) enrichment analysis on wheat, eight days post inoculation. Yellow background highlights calcium ion binding association. Thatcher*Lr14a* compared to Thatcher infected with an avirulent, a virulent or no leaf rust pathogen isolate. Statistics: (GoSeq), Wallenius non-central hypergeometric distribution and Benjamini-Hochberg method were used.

**Supplementary Data 4** Results of the RNAseq analysis in Thatcher/Thatcher*Lr14a* wheat after transient expression of *Lr14a*. Differentially expressed genes (DEG) associated with calcium ion binding under the three different conditions between Thatcher*Lr14a* and Thatcher, infected with an avirulent, a virulent or no leaf rust pathogen isolate. Statistics: (EdgeR), generalized linear models (GLMs), likelihood ratio test and Benjamini-Hochberg method were used.

**Supplementary Data 5** Results of the RNAseq analysis in Thatcher/Thatcher*Lr14a* wheat after transient expression of *Lr14a*. All Differentially expressed genes (DEG) between Thatcher*Lr14a* and Thatcher, infected with an avirulent, a virulent or no leaf rust pathogen isolate. Statistics: (EdgeR), generalized linear models (GLMs), likelihood ratio test and Benjamini-Hochberg method were used.

**Supplementary Data 6** Results of the RNAseq analysis in *Nicotiana benthamiana* after transient expression of *Lr14a*. Gene ontology (GO) enrichment analysis on *N. benthamiana*, 27 hours post inoculation with Agrobacteria, carrying an overexpression HA tagged *Lr14a* coding sequence respectively an empty vector comparison control. Yellow background highlights calcium ion binding association. Statistics: (GoSeq), Wallenius non-central hypergeometric distribution and Benjamini-Hochberg method were used.

**Supplementary Data 7** Results of the RNAseq analysis in *Nicotiana benthamiana* after transient expression of *Lr14a*. **e,** *N. benthamiana* differentially expressed genes (DEG), 27 hours post inoculation with Agrobacteria, carrying an overexpression HA tagged *Lr14a* coding sequence respectively an empty vector comparison control. Statistics: (EdgeR), generalized linear models (GLMs), likelihood ratio test and Benjamini-Hochberg method were used.

**Supplementary Data 8** Results of the RNAseq analysis in *Nicotiana benthamiana* after transient expression of *Lr14a*. All Differentially expressed genes (DEG) of *N. benthamiana* 27 hours post inoculation with Agrobacteria, carrying an overexpression HA tagged *Lr14a* coding sequence

respectively an empty vector comparison control. Statistics: (EdgeR), generalized linear models (GLMs), likelihood ratio test and Benjamini-Hochberg method were used.

**Supplementary Data 9** Primers used in this study. List of all primers used in this study for *Lr14a* marker analysis, *Lr14a* amplification, *Lr14a* sequencing, *Lr14a* VIGS constructs, *Lr14a* qPCR, *ADP-RF* qPCR, *PR1,2,5*[28] qPCR, *Lr14a* race PCR, *Lr14a* pENTR cloning, and Arina*LrFor Lr14a* region breakpoints.

**Supplementary Data 10** Results of 3000 bp MutChromSeq analysis. The MutChromSeq pipeline was run on synthetic assemblies where the pseudomolecule of chromosome 7B was split *in silico* into contigs of 3000 bp.

**Supplementary Data 11** Results of 5000 bp MutChromSeq analysis. The MutChromSeq pipeline was run on synthetic assemblies where the pseudomolecule of chromosome 7B was split *in silico* into contigs of 5000 bp.

**Supplementary Data 12** Results of 7000 bp MutChromSeq analysis. The MutChromSeq pipeline was run on synthetic assemblies where the pseudomolecule of chromosome 7B was split *in silico* into contigs of 7000 bp

**Supplementary Data 13** Results of MutChromSeq analysis. The results for contig size 3000 bp were screened visually for EMS mutations (mostly G/C to A/T). The three candidate contigs identified in this way were searched for genes by blast against IWGSC Reference Sequence v1.0 gene annotation.
